# A Yeast-Based Functional Assay to Study Plant N-Degron – N-Recognin Interactions

**DOI:** 10.3389/fpls.2021.806129

**Published:** 2022-01-07

**Authors:** Aida Kozlic, Nikola Winter, Theresia Telser, Jakob Reimann, Katrin Rose, Lilian Nehlin, Sophie Berckhan, Gunjan Sharma, Charlene Dambire, Tinne Boeckx, Michael J. Holdsworth, Andreas Bachmair

**Affiliations:** ^1^Max Perutz Labs, Department of Biochemistry and Cell Biology, University of Vienna, Vienna, Austria; ^2^School of Biosciences, University of Nottingham, Nottingham, United Kingdom

**Keywords:** synthetic biology, ubiquitin-dependent proteolysis, N-degron pathway, yeast-based assay, PRT6, N-recognin, *Arabidopsis thaliana*

## Abstract

The N-degron pathway is a branch of the ubiquitin-proteasome system where amino-terminal residues serve as degradation signals. In a synthetic biology approach, we expressed ubiquitin ligase PRT6 and ubiquitin conjugating enzyme 2 (AtUBC2) from *Arabidopsis thaliana* in a *Saccharomyces cerevisiae* strain with mutation in its endogenous N-degron pathway. The two enzymes re-constitute part of the plant N-degron pathway and were probed by monitoring the stability of co-expressed GFP-linked plant proteins starting with Arginine N-degrons. The novel assay allows for straightforward analysis, whereas *in vitro* interaction assays often do not allow detection of the weak binding of N-degron recognizing ubiquitin ligases to their substrates, and *in planta* testing is usually complex and time-consuming.

## Introduction

The ubiquitin-proteasome system for protein turnover is well conserved in all eukaryotes. In an enzymatic cascade, the ubiquitin carboxyl terminus is linked to a Cys residue of ubiquitin activating enzyme to form a thioester. From there, ubiquitin is transferred to the active site of a ubiquitin conjugating enzyme. With help of ubiquitin ligases, ubiquitin is transferred to the ε-amino groups of Lys residues (or, rarely, to OH groups of Ser or Thr) in protein substrates. Ubiquitin conjugation changes substrate properties and often leads to rapid degradation. For instance, N-recognins, i. e. ubiquitin ligases that recognize first amino acids in their substrates (so-called N-degrons), usually attach a ubiquitin chain that results in turnover of the substrate by a multi-subunit protease, the proteasome. While many aspects of ubiquitylation are conserved, species-specific features are also an inherent characteristic. In particular, the increasing complexity of multicellular organisms is mirrored in an increasing number of ubiquitin ligases and ubiquitin conjugating enzymes. This pertains also to the degradation routes for N-degrons. Whereas baker’s yeast *S. cerevisiae* has a single ubiquitin ligase, UBR1, with specificity for N-degrons consisting of an unmodified amino-terminal residue with large side chain, animals and plants have several N-recognins with this specificity.

Plants have distinct substrates and possess several N-recognins that do not occur in animals or in fungi (Dissmeyer, 2019; Millar et al., 2019; Holdsworth et al., 2020). Even the major conserved N-recognin, called PROTEOLYSIS6 (PRT6), differs in substrate specificity from its yeast and mammalian homologs, UBR1 and Ubr1/Ubr2, respectively (Garzon et al., 2007). For an in-depth description of N-recognin dependent turnover, it is therefore necessary to investigate species-specific enzymes and substrates. This task is, however, particularly challenging for plants, due to the lack of fast assays. A possible solution to this problem is to circumvent analysis in the natural host.

One approach uses plant extracts, to which potential substrates can be added (Takahashi et al., 2009; Ramadan et al., 2015). However, these systems are poorly suited for dissection of degradation pathways, even though preparation of extracts from mutant plants can be instructive. *In vitro* reconstitution has been used extensively for many enzymes of ubiquitin conjugation. Modification of potential substrates could often be shown (Stone et al., 2005; Zhao et al., 2013), in addition to auto-ubiquitylation of most E3 ligases under these conditions. However, a functional link to proteasomal degradation is not part of most *in vitro* systems, and quantitative aspects of ubiquitylation or turnover can be investigated only in exceptional cases (Mot et al., 2018). Regarding ubiquitin conjugation, heterologous expression in *E. coli* has also been applied successfully in various instances (Han et al., 2017; Kowarschik et al., 2018). Heterologous *in vivo* expression in *E. coli* does not require enzyme purification, which often speeds up experiments and may allow higher throughput setups. However, a natural limit of *E. coli* is that, with its prokaryotic folding catalysts, expression of large eukaryotic proteins may come to a limit. We have therefore considered baker’s yeast *S. cerevisiae* as a heterologous host for assaying ubiquitylation enzymes. Yeast has a single N-recognin for unmodified N-termini with large side chains, Ubr1, and deletion of UBR1 generates a background where these N-degrons are no longer destabilizing. N-recognins from other organisms (in our case, from plants) can then be analyzed by co-expression with additional proteins including ubiquitin conjugating enzymes and substrates, to assess functional competence. The strategy has previously been used to elucidate the substrate specificity of plant N-recognin PRT1 (Stary et al., 2003). In this setup, the functional relevance of ligase – substrate interactions can be studied even if affinities are too weak for standard interaction assays such as co-immunoprecipitation or yeast two hybrid assay.

In a synthetic biology approach to study N-degron – N-recognin relations, we thus established an N-degron turnover pathway in baker’s yeast based on plant N-recognin PRT6. After successful use of our classical model substrate β-Gal, we introduced another model substrate based on GFP, and we tested the system with confirmed and potential substrates from Arabidopsis.

## Materials and Methods

### Yeast Strains

Yeast strain CB80 (Mat *a ura3-52 leu2-3*,*112 trp1-1 his3-*Δ*200* Gal^+r^) was transformed with a KanMX cassette (Wach et al., 1994) flanked by DNA from the UBR1 locus. In effect, the section from nt −26 to nt 5387 was replaced by the G418 resistance cassette, to obtain yeast strain CB80 Δ*ubr1* (Mat *a ura3-52 leu2-3*,*112 trp1-1 his3-*Δ*200* Δ*ubr1* Gal^+^, G418^r^).

### Vector Cloning

Vectors for Expression of βGal substrates have been described (Bachmair et al., 1986; pUB23 of Addgene), an annotated sequence can be found as Supplementary File 1. Vector YEpURAubXGFP was assembled from the backbone YEplac195 (Gietz and Sugino, 1988), supplied with promoter- and terminator region from vector pAG413GAL-ccdB (Addgene), to express yeast ubiquitin followed in frame by a superfolder-GFP cassette (Zhang et al., 2019). Between the ubiquitin and GFP sequence part is a unique *Sfo*I site that allows insertion of ORFs that are positioned amino-terminally upon ubiquitin cleavage in yeast. The sequence of the vector can be found in Supplementary File 2.

cDNAs for potential PRT6 substrates were cloned from isolated mRNA. Constructs to fuse model substrates or Arabidopsis ORFs with the fluorescent protein were generated after *Sfo*I cleavage of the vector and PCR amplification of the ORF to be inserted, using In-Fusion cloning (TaKaRa). Insert sequences are listed in Supplementary File 3.

The ORF of Arabidopsis PRT6 was isolated from reverse-transcribed mRNA and first cloned into the low copy vector pACYC177 (between *Bam*HI and *Aat*II sites). From there, the ORF was inserted into expression vector YCplac22 (Gietz and Sugino, 1988) equipped with phosphoglycerate kinase promoter and terminator cloned from vector pMA91 (Mellor et al., 1983). This YCplac22-PGK promoter expression vector was previously used to express N-recognin PRT1 in yeast (Stary et al., 2003). In order to express, in addition to PRT6, Arabidopsis UBCs in yeast, vector pAG413GAL-ccdB (Addgene) was employed. This vector carries a HIS3 marker and the inducible GAL10 promoter. The Arabidopsis AtUBC2 cDNA was PCR-amplified from a vector designed for expression in *E. coli* (Kowarschik et al., 2018). Sequences of the vectors for expression of PRT6 and AtUBC2 can be found in Supplementary Files 4, 5, respectively.

### Protein Quantitation

For β-galactosidase substrates, protein extracts were prepared from cultures inoculated with freshly transformed yeast colonies. These were suspended in synthetic medium containing 2% glucose and grown overnight at 30°C. Aliquots were transferred into medium containing 2% galactose and 0.1% glucose. After 4.5 h growth, these cultures were diluted in 15 ml selective medium with 2% galactose so that O/N growth resulted in an OD600 of 0.8. Extracts were prepared from cell pellets of 10 ml of culture by shaking with glass beads (FastPrep24 bead beating grinder) in buffer (100 mM TrisHCl pH 8, 20% glycerol, 1 mM dithiothreitol, 1 mM phenylmethylsulfonyl fluoride). Cleared extracts were used for determination of protein content (Protein Assay Dye reagent, BioRad), and for β-galactosidase activity using ortho-nitrophenyl-β-D-galactoside (ONPG, Sigma; 4 mg/ml dissolved in Z buffer) as a substrate, by incubating 100 μl of extract with 900 μl of Z buffer at 28°C. After addition of 200 μl ONPG solution, extracts were incubated and the reaction was stopped by addition of 0.5 ml 1 M Na_2_CO_3_. Optical density at 420nm (OD420) was normalized to incubation time and protein content.

For fluorescent proteins, cultures adapted to galactose medium were diluted to an OD600 of 0.3 and distributed into 96 well microtiter plates. Samples encompassed both biological (independently transformed yeast colonies) and technical replicates. In a microtiter plate reader (BioTek Synergy H1), GFP emission at 515 nm was measured after excitation at 485 nm. In addition, OD600 and path length were recorded to obtain values for cell density determination.

### Stability Assay *via* Rabbit Reticulocyte Lysate

*In vitro* stability analysis of MetCys-initiating proteins was carried out using a rabbit reticulocyte lysate (RRL) assay, that contains all the components of the Arg/N-degron pathway, as described in Gibbs et al. (2011, 2018). Coding regions were amplified from Arabidopsis seedling cDNA and cloned into a modified pTNT expression vector (Invitrogen) containing a C-terminal 3X-HA tag providing expression under the T7 promoter. C2A substitutions were carried out through site-directed mutagenesis. *In vitro* translation was done for 30 min at 30°C using Promega TNT ^®^ Coupled Rabbit Reticulocyte Lysate System as per manufacturer’s instructions. Reaction mixes with constructs containing MC-initiating proteins were set up with or without 50 μM MG132/bortezomib. After 30 min incubation, 1 μl of 2.6 mM cycloheximide (final concentration 173 μM) was added and samples were collected at indicated time points. Western blots were carried out as previously described (Gibbs et al., 2018). Anti-HA primary antibody (Sigma, 1:2500), anti-actin (Sigma, 1:1000), anti-tubulin (Sigma, 1:5000) and anti-mouse or anti-rabbit secondary antibodies (Santa Cruz, 1:10,000 dilution) were used to detect HA-tagged and internal control proteins. ECL western blotting chemiluminescent detection kit (GE Healthcare) was used to develop blots.

### Western Blotting of Yeast Extracts

Yeast cells were washed, pelleted, and resuspended and boiled in 1 x Laemmli sample buffer to obtain extracts. After transfer, blots were developed using mouse anti-GFP antibody (Roche, 1:1000 dilution) followed by HRP-conjugated goat anti-mouse antibody (Cytiva NA931, 1:10 000 dilution) and Western Bright chemiluminescent detection reagent (advanstra). ChemiDoc MP (BioRad) was used for detection.

## Results and Discussion

### Arabidopsis N-Recognin PROTEOLYSIS6 Is Active in *S. cerevisiae* If Co-expressed With Ubiquitin Conjugating Enzyme 2

In order to re-constitute plant N-degron dependent turnover in baker’s yeast, we generated a yeast strain lacking the classical Arg/N-degron pathway by deletion of UBR1, the single yeast N-recognin for unblocked amino-terminal residues with large side chains. Yeast strain CB80 Δ*ubr1* and its parent CB80 were used as isogenic pair in this work.

**FIGURE 1:**
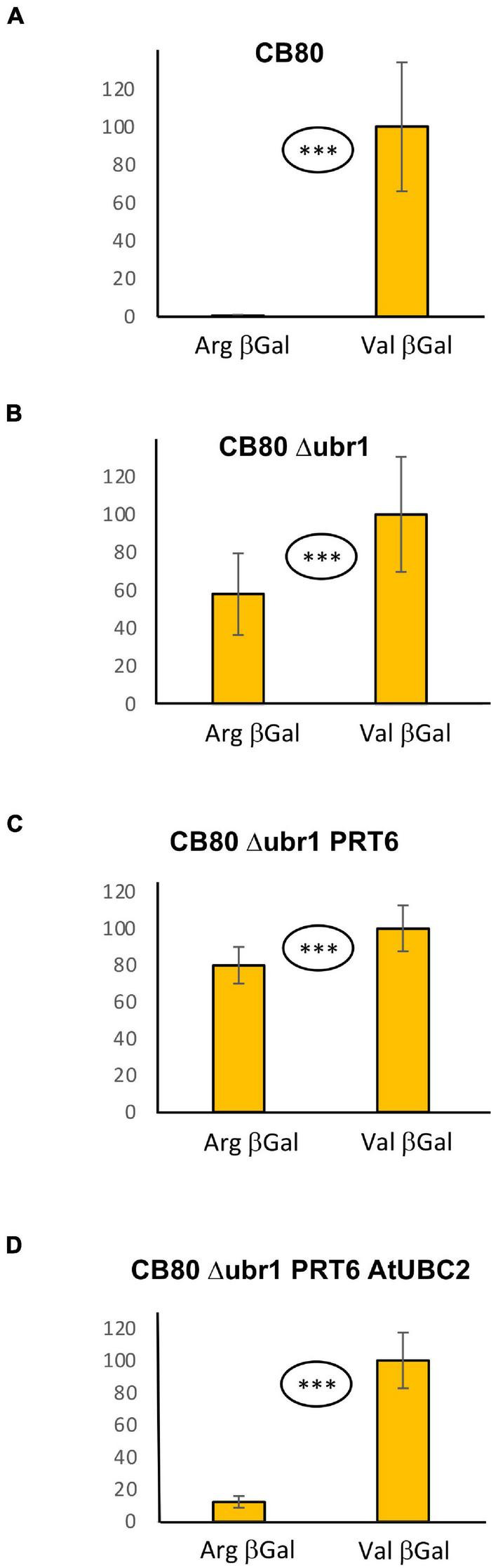
Steady-state levels of βGal-based model substrates starting either with Arg or with Val in different yeast strains **(A–D)**. Values of Val βGal were set 100%. Differences between columns are significant (^***^p < 0.001, two-sided *t* test). At least eight replicates were used for each colummn. Numerical values are listed in Supplementary File 6.

In the WT yeast strain CB80, Arg-βGal (Bachmair et al., 1986) is highly unstable and therefore much less abundant than Val-βGal (Figure 1A; 0.6% vs. 100%; for exact values, see Supplementary File 6). In CB80 Δ*ubr1*, which has the single yeast N-recognin UBR1 deleted, the Arg-βGal model substrate has 58% the abundance of Val-βGal (Figure 1B). Both proteins are expressed as fusions with amino-terminal ubiquitin (see Supplementary File 1). Upon co-translational cleavage of ubiquitin by ubiquitin-specific proteases, the test protein is released (Bachmair et al., 1986). When PRT6 was introduced into CB80 Δ*ubr1*, N-degron model substrate Arg-βGal was not less abundant than in the strain without PRT6 (Figure 1C). This indicated that PRT6 was not active. We hypothesized that no yeast ubiquitin conjugating enzyme (E2) was able to cooperate optimally with PRT6, and that this N-recognin therefore required an E2 from Arabidopsis. We found that expression of AtUBC2, in addition to PRT6, resulted in an active enzyme able to confer a short half-life on Arg-βGal (Figure 1D). Interestingly, AtUBC2 belongs to the Rad6 group of UBCs, which are involved in epigenetic control of gene expression. The yeast enzyme Rad6 is apparently not able to efficiently serve PRT6, but is the known E2 for the yeast PRT6 homolog Ubr1. Thus, while the E2-E3 pair is conserved, changes in one or both partners largely prevent activity of the mixed pair PRT6-Rad6.

### A New Vector for Testing of Arabidopsis Proteins

**FIGURE 2:**
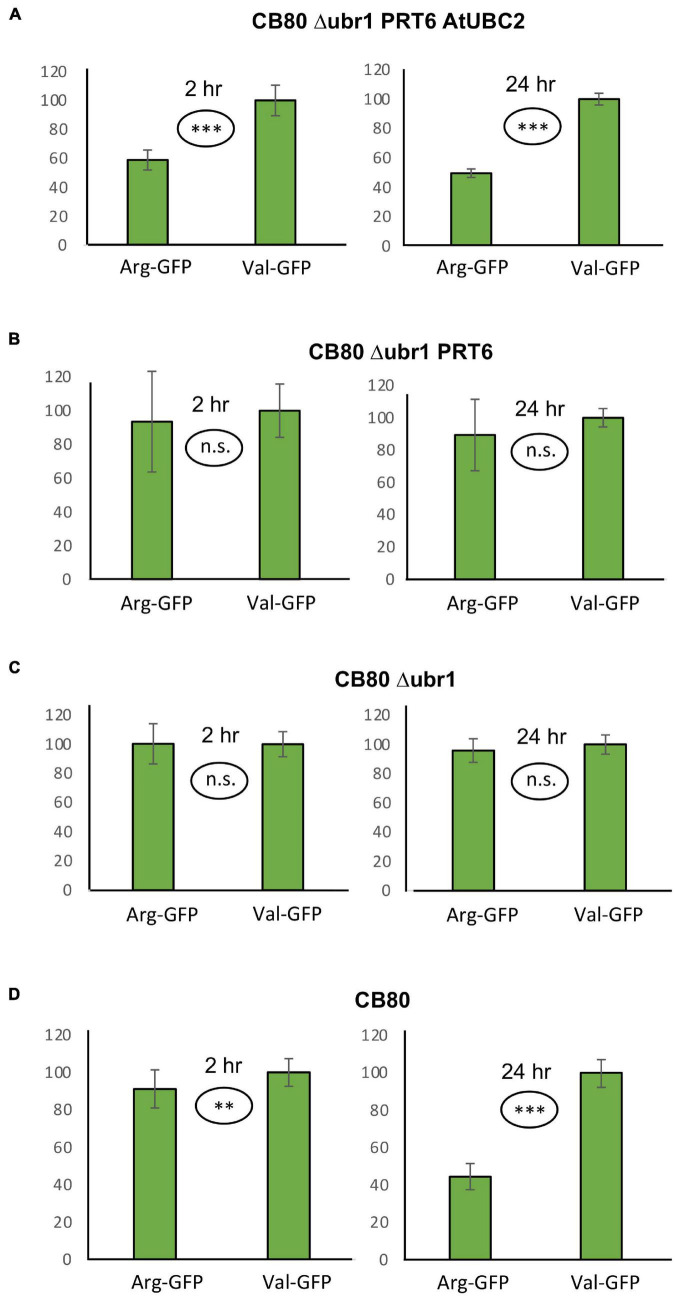
Steady-state levels of GFP-based model substrates starting either with Arg or with Val in different yeast strains. Mean value of Val-GFP was set 100%. Measurements with plate reader were carried out 2 h (right panels) and 24 h (left panels) after inoculation of microtiter wells. Plates were kept at 30°C to allow yeast growth. Differences in panel **(A)** (both time points) and panel **(D)** (24 h time point) are highly significant (^***^p < 0.001, two-sided *t* test). Difference in panel **(D)** (2 h time point) is significant (^**^p < 0.01, two-sided *t* test). Differences in panels **(B,C)** are not significant (n.s. *p* > 0.05, two-sided *t* test). At least fourteen replicates were used for each column. Numerical data are listed in Supplementary File 6.

The measurement of β-galactosidase activity to determine the model substrate steady state level is straightforward and inexpensive, but relatively time-consuming and, due to the considerable workload, not suitable for high throughput assays. We therefore designed a vector that should allow quantitation of protein abundance *via* fluorescence readout. When the vector YEpURAubXGFP was transformed into CB80 Δ*ubr1*, yeast cells expressed GFP when growing on galactose medium. We inserted a sequence that highly resembles the amino-terminal region of model substrates previously used in plants (for sequence, see Supplementary File 3; Garzon et al., 2007). Comparison of this GFP-based test protein resulted in a large difference of steady-state levels depending on the first residue (Arg vs. Val, Figure 2A). The difference largely disappears in the absence of AtUBC2 (Figure 2B), and in absence of both AtUBC2 and PRT6 (Figure 2C). For the sfGFP-based vector, the steady state level can be determined with a plate reader (excitation wavelength 485 nm, emission wavelength 515 nm) in whole cells. Cell cultures were allowed to grow for up to 24 h in the wells of a microtiter plate, the selective yeast growth medium being almost transparent. For each of these cultures, OD600 was also determined *via* plate reader, as a measure of the number of yeast cells present in the well. Cell density was used to compensate for differences in inoculation or growth in the wells. Figure 2 shows results with the new set of model substrates, Arg-GFP and Val-GFP, in different genetic backgrounds. Clearly, Arg-GFP is a somewhat poorer substrate than Arg-βGal, because its steady state level differs less from the otherwise identical protein starting with Val. An interesting detail is shown in Figure 2D. In WT yeast cells relying on N-recognin Ubr1, Arg-GFP is less abundant than Val-GFP by only a margin (91 per cent vs. 100 per cent) in mid-log cells. While this difference is significant at the 99% level (two-sided *t* test), it is surprisingly small. After 24 h, when cells reach late log or stationary phase, the difference is comparable to cells expressing PRT6 and AtUBC2 (Figures 2D vs. 2A). We suggest that Ubr1 activity increases when cells reach stationary phase, whereas PRT6 expression driven by the phosphoglycerate kinase promoter is high under all growth conditions. We finally carried out a Western blot experiment to assess whether endoproteolytic processing results in a pool of GFP that contains no N-degron. Such a pool could decrease differences between Arg-GFP and Val-GFP regarding steady-state abundance. As shown in Supplementary Figure 1, the majority of proteins cross-reacting with anti-GFP antibody has the size of full-length X-GFP in all genotypes tested. In total, we conclude that the GFP substrate vector allows qualitative assessment of N-degrons in the CB80 Δ*ubr1* PRT6 AtUBC2 yeast strain. In order to draw quantitative conclusions, additional tests such as pulse chase experiments to directly observe turnover kinetics, will be necessary in the future.

### Test of Arabidopsis cDNAs From the MC-ome

**FIGURE 3:**
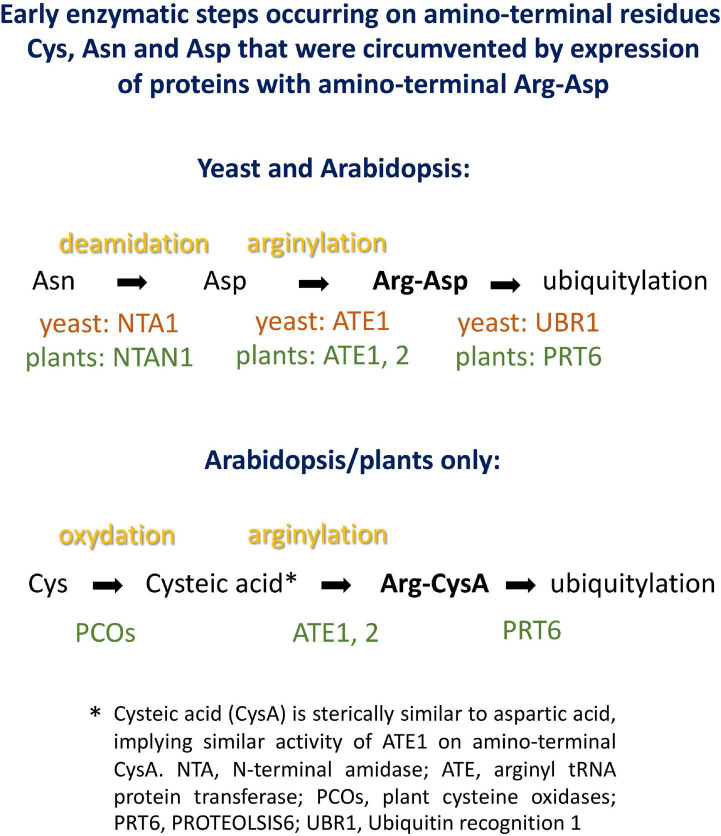
Potential processing steps occurring on amino-terminal Asn, Asp and Cys to generate substrates for N-recognins PRT6 and UBR1.

Plant proteins starting with Met-Cys (the “MC-ome”) lose their initiator methionine by the action of methionine aminopeptidase. Thereafter, the fate of the protein depends on several processing enzymes, and on the presence of molecular oxygen. Plant cysteine oxidases can, provided that the concentration of oxygen is high enough, oxidize Cys to cysteic acid. After oxidation, Arg tRNA protein transferase adds an Arg residue to the amino terminus. These maturation steps are listed in Figure 3. The processed protein may then be a substrate for PRT6-mediated ubiquitylation. For some proteins of this class, an oxygen-dependent rapid turnover, as well as dependence on PRT6, has already been demonstrated in plants. However, for others, this test could not yet be carried out. Because an *in planta* assay requires considerable resources and time, we used the yeast system to obtain evidence regarding turnover *via* PRT6 in yeast. Known substrates expressed in yeast were compared to the existing body of *in planta* data, and untested proteins were expressed to make predictions about potential *in planta* stability. Because the assay proposed focuses on the N-recognin, we circumvented the processing steps by expressing proteins with amino-terminal Arg-Asp instead of Met-Cys (Asp is structurally similar to oxidized Cys, and Arg is posttranslationally appended to acidic first residues; cf. Figure 3). These proteins were also compared to the same ORFs starting with Ala-Asp. Similar to Val, Ala is a stabilizing residue. It was previously used for *in planta* stability assays, and a first residue Ala makes the results directly comparable to published results. Table 1 lists the proteins chosen for testing. ORFs were cloned into the sfGFP vector (for sequences, see Supplementary File 3).

**TABLE 1:** Arabidopsis proteins investigated as fusions to GFP for stability in modified yeast cells.

Protein name	Identifier	Presumed metabolic instability in		
		Arabidopsis	Yeast	Reticulocyte lysate
HRE2	At2g47520	Yes [1]	Yes	Yes
VRN2	At4g16845	Yes [2]	No*	Yes
ZPR2	At3g60890	Yes [3]	Yes	No
BBX31	At3g21890	n. t.	Yes	Yes
BBX30	At4g15248	n. t.	Yes	Yes
bHLH38	At3g56970	n. t.	No**	Yes
RIN4 fragm. II	(At3g25070)	Yes [4]***	No	n. t.
RIN4 fragm. III	(At3g25070)	Yes [4]****	±	n. t.

*[1] Gibbs et al. (2011); [2] Gibbs et al. (2018); [3] Weits et al. (2019); [4] Goslin et al. (2019).*

*Low abundance of full-length protein thwarts assay.

**Construct may be subject to turnover independent of PRT6, as it is also unstable in a yeast strain without AtUBC2.

***Involvement of PRT6 unlikely.

****Involvement of PRT6 probably redundant with other pathway(s).

*±, weak evidence for PRT6-dependent turnover; n.t., not tested.*

**FIGURE 4:**
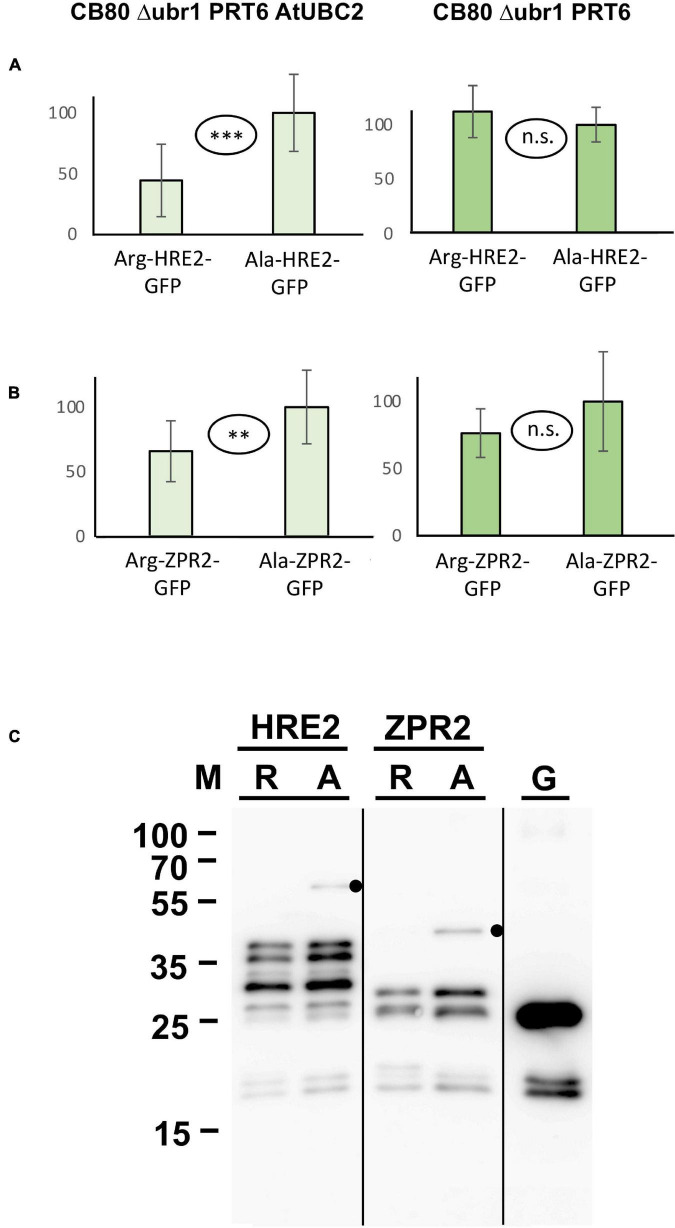
Steady-state levels of Arabidopsis substrates of PRT6 in different yeast strains. The GFP fusions with HRE2 and ZPR2, respectively, start either with Arg or with Ala (see Figure 3 and text for amino-terminal processing, and Supplementary File 3 for amino acid sequences). **(A,B)** Mean abundance of the proteins starting with Ala was set 100%. Standard deviations are shown as error bars. Measurements were carried out 2 h after inoculation of microtiter wells. Plates were kept at 30°C to allow yeast growth. Differences are indicated as highly significant (^***^p < 0.001, two-sided *t* test), significant (^**^p < 0.01, two-sided *t* test) and not significant (n.s. *p* > 0.05, two-sided *t* test). Twelve replicates were used for each column. Numerical data are listed in Supplementary File 6. **(C)** Western blot detection of GFP fusion proteins starting with Arg (R) or with Ala (A). Extracts were taken from yeast strain expressing PRT6 and AtUBC2. Note increased level of the full-length proteins starting with Ala. Unfused GFP (G) is shown in the right lane. Dots indicate expected positions of full length fusion proteins.

N-degron dependent turnover of the three proteins HRE2, VRN2 and ZPR2 in Arabidopsis has been reported (Gibbs et al., 2011, 2018; Weits et al., 2019). Figure 4 demonstrates that Arg-HRE2-GFP and Arg-ZPR2-GFP show robust N-degron dependent turnover, both regarding measurement of GFP activity, and detection of the protein *via* anti-GFP Western blotting. Absence of AtUBC2 abrogates the difference between fusion proteins starting with Arg and Ala, indicating N-degron dependence (Figures 4A,B). Western blotting (Figure 4C) indicates that the full-length protein starting with Ala is detectable, whereas the same protein with amino-terminal Arg is much less abundant and was not detected on the blot. The anti-GFP antibody also detects a number of smaller bands interpreted as proteolytic fragments derived from the full-length protein. These also show differential abundance and probably contribute to the GFP fluorescence signal. By contrast, VRN2 did not give conclusive results (Supplementary Figure 2A). A Major factor in the latter case seems to be that the fusion protein is generally unstable in yeast, so that GFP levels are low (for absolute GFP values, see Supplementary File 6). Interestingly, Western blotting still shows differential presence/accumulation of an apparently full-length protein with first residue Ala vs. the Arg version (Supplementary Figure 2B).

**FIGURE 5:**
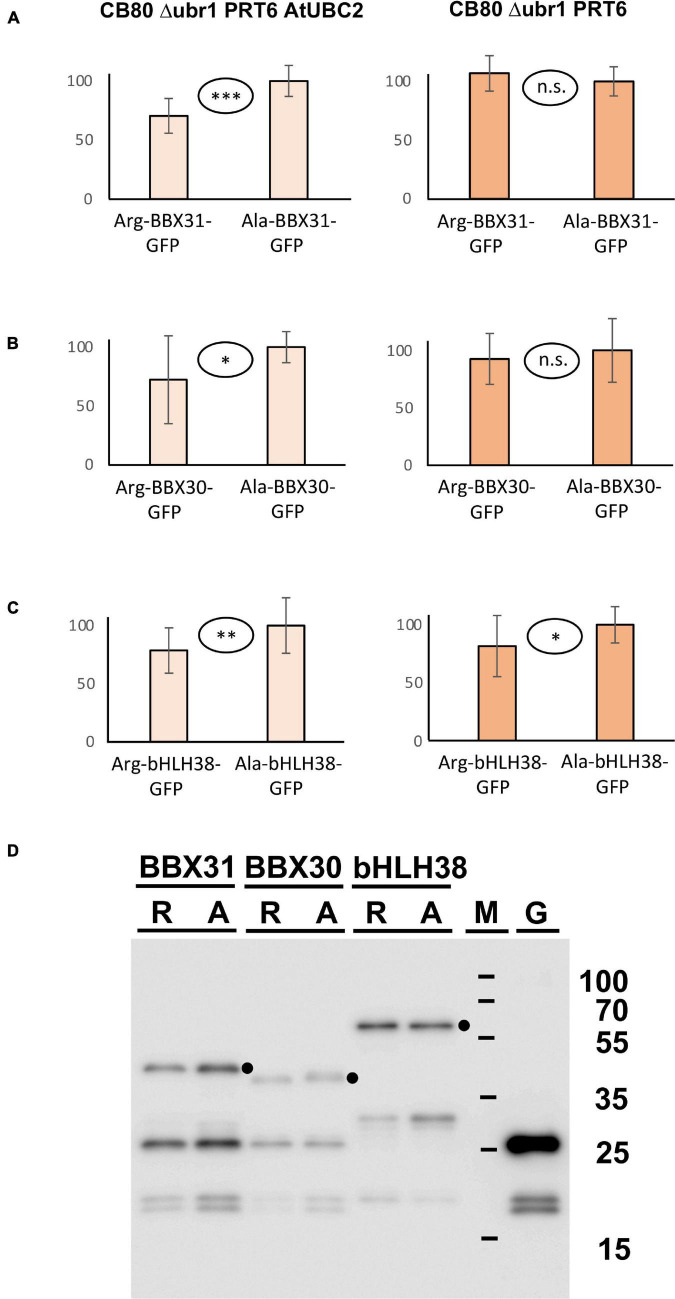
**(A–C)** Steady-state levels of Arabidopsis proteins BBX31, BBX30, and bHLH38 fused to GFP in different yeast strains. The GFP fusions start either with Arg or with Ala (see Figure 3 and text for amino-terminal processing, and Supplementary File 3 for amino acid sequences). Mean abundance of the protein starting with Ala was set 100%. Standard deviations are shown as error bars. Measurements were carried out 2 h after inoculation of microtiter wells. Plates were kept at 30°C to allow yeast growth. Differences are classified by statistical significance (^***^p < 0.001; ^**^p < 0.01; *p < 0.05; n.s., not significant, *p* > 0.05; two-sided *t* test). At least nine replicates were used for each column. Numerical data are listed in Supplementary File 6. **(D)** Western blot with anti-GFP antibody indicates that in all cases, the fusion protein is abundant and detectable. Extracts were taken from a yeast strain expressing PRT6 and AtUBC2. M, marker lane; G, extract expressing unfused GFP. Dots indicate positions of full length fusion proteins.

**FIGURE 6:**
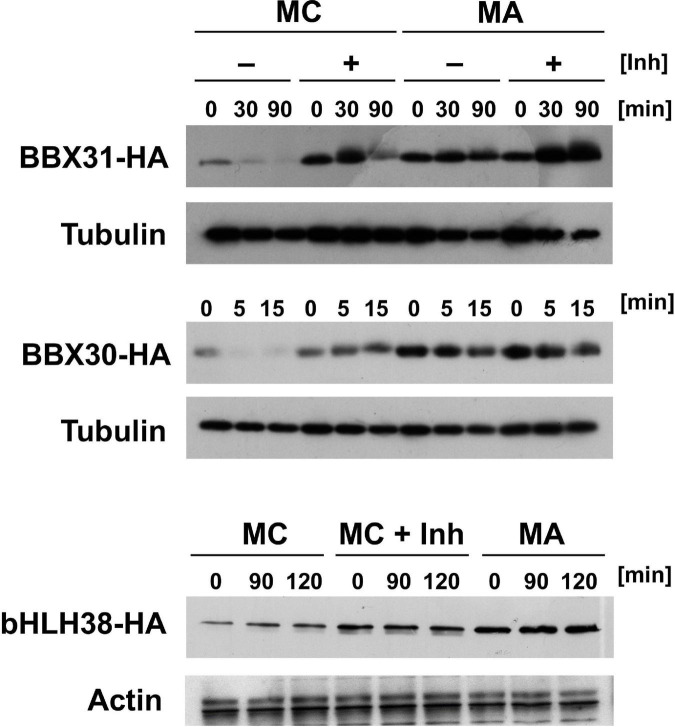
Time course experiments with Arabidopsis proteins BBX31, BBX30 and bHLH38 fused to the HA tag in rabbit reticulocyte lysates. Proteins were expressed starting with either Met-Cys (MC), or with Met-Ala (MA; see Figure 3 and text for amino-terminal processing that results in proteins starting either with Arg or with Ala). Detection was carried out by Western blot using anti HA antibody, and anti-tubulin or -actin as a loading control. Inh indicates presence of a proteasome inhibitor. Time of sampling after stop of translation by cycloheximide addition is indicated in minutes (min).

In addition to the three proteins with *in planta* resolved turnover, we tested one bHLH transcription factor that starts with Met-Cys (bHLH38, At3g56970), and the two closely related small proteins BBX30 and BBX31 (At4g15248 and At3g21890, respectively). Pfam assigns a single Zinc finger-like B-BOX domain to BBX30, whereas this same domain was not identified in BBX31. All of these ORFs are relatively short and probably serve as nuclear DNA- or chromatin-binding proteins. Figure 5A–C shows the results obtained with these proteins. For the BBX proteins, the yeast assay suggests that they are substrates of PRT6 (for BBX30, the assay was statistically less convincing than for BBX31). One surprising finding was that for bHLH38, omission of AtUBC2 from the yeast host did not lead to equal steady state levels of the GFP fusion proteins starting with Arg and Ala. Instead, there was still a difference between the Arg- and the Ala-version. We have currently no explanation for this result, but it may mean that an endogenous yeast turnover pathway obscures any specific involvement of PRT6, so that the assay remains inconclusive. Western blotting (Figure 5D) shows that all fusion proteins are present at detectable levels, consistent with the aim that most GFP fluorescence comes from the fusion protein, not from smaller proteolytic fragments that do not contain the N-degron up for testing. While the stability of these proteins in Arabidopsis has yet to be determined, data were collected after expression in a rabbit reticulocyte lysate. Figure 6 shows Western blots of samples, using proteins BBX31, BBX30 and bHLH38 linked to HA tag, starting with Met-Cys or with Met-Ala. In the cell-free reticulocyte extract, conditions allow oxidation of Cys and subsequent arginylation (cf. Figure 3), and differential accumulation of the two amino-terminal variants indicates N-degron dependence. As a further tool, addition of proteasome inhibitor demonstrates metabolic instability. Moreover, sample withdrawal at different time points after inhibition of translation by cycloheximide can directly demonstrate metabolic instability. In this assay, all three proteins are less abundant with a second residue Cys, which indicates that they are substrates of N-degron-dependent turnover.

Expression of proteins in yeast starting with Arg-Asp- instead of the Met-Cys- sequence encoded in the Arabidopsis genome side-steps modification reactions that might, in plants, be rate-limiting or substrate-selective. It seems possible to reconstitute these steps in yeast, as well. In particular, an oxidation step based on expression of plant enzymes converting amino-terminal Cys into cysteic acid has already been shown in yeast (Puerta et al., 2019). Regarding posttranslational linkage of Arg to the oxidized Cys terminus, yeast has an Arg-tRNA protein transferase that could, at least in certain cases, step in to modify Arabidopsis proteins. However, replacement of *S. cerevisiae* ATE1 by *A. thaliana* ATE1 coding sequence might allow to study additional features, similarities and differences between the two enzymes. AtATE1 activity has been demonstrated *in vitro* (White et al., 2017), so that there is no reason to assume that the enzyme would not work in the yeast cytoplasm or nucleus. Thus, the system developed in this work could be extended to encompass Arabidopsis enzymes for the entire “Met-Cys” pathway, to process proteins starting with Met, or after Met aminopeptidase removes the first translated residue (cf. Figure 3).

### Substrates With Unresolved Degradation Pathways in Arabidopsis

Basal immunity factor RIN4 is a peripheral membrane protein and target of several bacterial effectors (Mackey et al., 2003). One effector, AvrRpt2, cleaves RIN4 at two positions. The exposed amino termini are Asn (fragment RIN4-II) and Asp (fragment RIN4-III). Enzymes have been described that convert Asn termini into Asp termini, and Arg-tRNA protein transferases then produce an Arg-Asp protein. After amino-terminal processing, these fragments are therefore potential substrates for PRT6. As for the Met-Cys proteins, we circumvented amino-terminal processing steps (Figure 3), in order to focus on the contribution of PRT6 to turnover.

**FIGURE 7:**
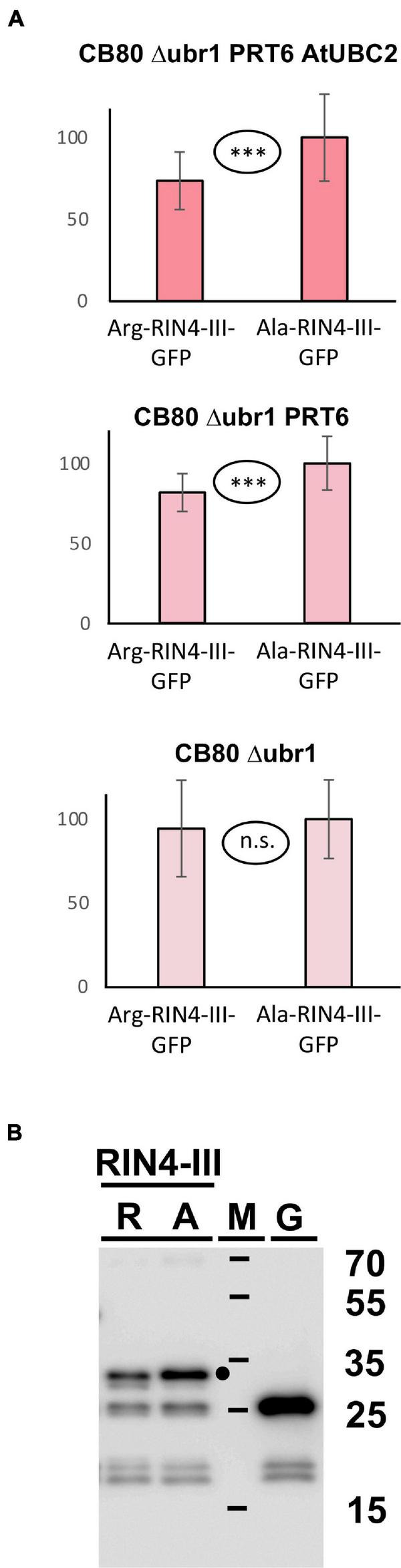
**(A)** Steady-state levels of Arabidopsis protein fragment RIN4-III fused to GFP in different yeast strains. The protein starts either with Arg, or with Ala (see Figure 3 and text for protein processing steps, Supplementary File 3 for amino acid sequences and Supplementary File 6 for numerical data). Mean abundance of the protein starting with Ala was set 100%. Standard deviations are shown as error bars. Measurements were carried out 2 h after inoculation of microtiter wells. Plates were kept at 30°C to allow yeast growth. Differences are indicated as highly significant (^***^p < 0.001, two-sided *t* test), significant (^**^p < 0.01, two-sided *t* test), or not significant (n.s. *p* > 0.05, two-sided *t* test). **(B)** Western blot detection of GFP fusion proteins starting with Arg (R) or with Ala (A). Extracts were taken from yeast strain expressing PRT6 and AtUBC2. M, marker lane; G, extract expressing unfused GFP. Sizes of marker proteins are indicated to the right. Dot indicates position of full-length protein.

Goslin et al. (2019) found that the larger fragment RIN4-II obtained after AvrRpt2 cleavage is not stabilized in a mutant devoid of amino-terminal arginylation (Arabidopsis *ate1 ate2* mutant), and for RIN4-III the *in planta* turnover status could also not be resolved, although a slight stabilization in the *ate1 ate2* background could be detected for a fusion protein containing this fragment at amino-terminal position (Goslin et al., 2019). Generally, the *in planta* data suggested that these two fragments are either not substrates of the N-degron pathway, or that more than one pathway operates in parallel. We employed the yeast synthetic biology approach to find out whether PRT6 can use any of these fragments as a substrate, provided processing by N-terminal amidase and Arg tRNA protein transferase operates. Our experiments showed no significant difference between the Arg- and the Ala- versions of the RIN4-II fragment (data not shown). This is consistent with the published results that this fragment is not a good *in vivo* substrate for PRT6 (Goslin et al., 2019). In our assay (Figure 7A), the RIN4-III fragment is substrate of PRT6 in yeast, with differential stability of the Arg- vs. the Ala-protein. Interestingly, as previously found for bHLH38, the assay shows that PRT6 without AtUBC2 also results in an abundance difference between the Arg- and the Ala version. When the protein pair is expressed in yeast CB80Δubr1, this difference apparently disappears. One explanatory hypothesis is that the RIN4-III fragment can help to recruit a yeast E2 to PRT6, but more experiments are needed to confirm this interpretation. The experiment certainly suggests that yeast strain CB80 Δ*ubr1* is probably a better control strain than CB80 Δ*ubr1* containing PRT6 (as used in previous Figures). Western blotting (Figure 7B) indicates that the full-length protein is more abundant than any proteolytic fragment.

### Comparison to the Use of Rabbit Reticulocyte Extract

Proteins expressed in a combined transcription-translation system generated from reticulocytes (cf. Figure 6) were previously used as a pre-screening option to identify proteins from the over 200 ORF strong MC-ome of Arabidopsis that are potential N-recognin substrates. Most of the proteins used in this work were also tested in this experimental setup (Table 1). These experiments used proteasome inhibitor and sampling in a pulse chase scheme to specifically demonstrate metabolic instability. In contrast, the yeast system relies on different genetic backgrounds of the yeast host (PRT6 plus AtUBC2 vs. absence of at least one of these enzymes). Furthermore, the steps of Cys oxidation and arginylation are side-stepped in the yeast system. To summarize, the yeast system introduced in this work is in some aspects complementary to the retic system (for instance, for VRN2) and seems to be a useful predictor of metabolic instability in plants that may be easier to handle than a rabbit reticulocyte extract.

## Conclusion

We show that a synthetic biology approach to set up protein ubiquitylation of plant and model substrates by plant E2/E3 pairs is possible for the PRT6/N-degron pathway and leads to subsequent degradation of the substrates in yeast. In several cases, we observe presumed basal turnover activity of the control protein without destabilizing first residue. However, proper controls (expression of the same protein in a strain lacking either PRT6 or AtUBC2 or both) allowed us to demonstrate the contribution of PRT6 to turnover. It is possible that mutants in well-known yeast quality control ligases such as SAN1 (Hampton, 2011) could improve the differential between N-degron substrates and non-substrates, by eliminating competing pathways of turnover in yeast. However, modified genetic backgrounds may not be necessary, as long as the contribution of the heterologously expressed ligase is statistically significant. In this sense, yeast as a synthetic biology host may even be interesting for testing non-N-degron metazoan ligases together with their potential substrates in a ± ligase comparison.

## Data Availability Statement

The original contributions presented in the study are included in the article/Supplementary Material, further inquiries can be directed to the corresponding author.

## Author Contributions

AK, NW, TT, JR, KR, LN, CD, TB, SB, and GS carried out the experiments. NW, MJH, and AB designed the experiments and contributed to manuscript writing. All authors contributed to the article and approved the submitted version.

## Conflict of Interest

The authors declare that the research was conducted in the absence of any commercial or financial relationships that could be construed as a potential conflict of interest.

## Publisher’s Note

All claims expressed in this article are solely those of the authors and do not necessarily represent those of their affiliated organizations, or those of the publisher, the editors and the reviewers. Any product that may be evaluated in this article, or claim that may be made by its manufacturer, is not guaranteed or endorsed by the publisher.
