# Supplementary material for: A Yeast-Based Functional Assay to Study Plant N-Degron – N-Recognin Interactions
Source: Front Plant Sci. 2022 Jan 7;12:806129. doi: 10.3389/fpls.2021.806129 (PMC8777003; doi:10.3389/fpls.2021.806129)
Supplement: Supplementary file 1 [file Image_1.pdf]

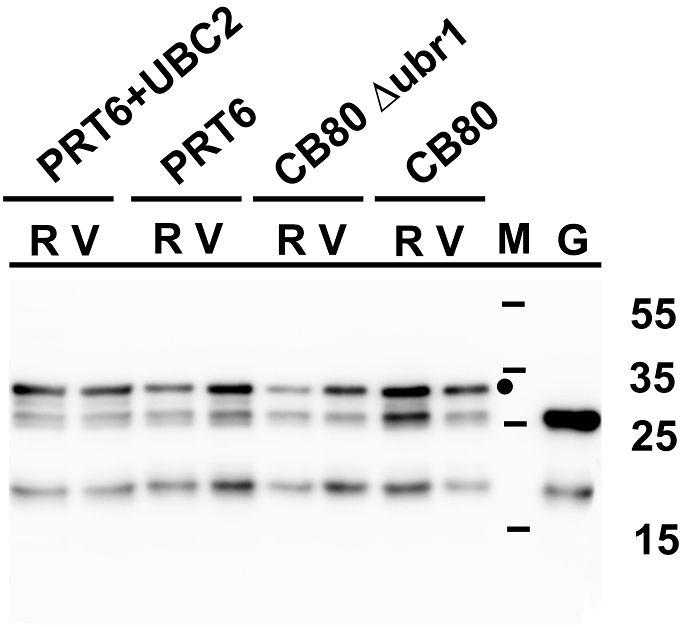

Supplementary Figure 1. Anti-GFP Western blot to assess nonspecific cleavage of Arg-GFP (R) and Val-GFP (V) constructs in different genetic backgounds. M, marker Lane (values to the right). G, extract from yeast cells expressing GFP vector without extension. Dot indicates expected position of full length fusion proteins.
