# Supplementary material for: A Yeast-Based Functional Assay to Study Plant N-Degron – N-Recognin Interactions
Source: Front Plant Sci. 2022 Jan 7;12:806129. doi: 10.3389/fpls.2021.806129 (PMC8777003; doi:10.3389/fpls.2021.806129)
Supplement: Supplementary file 2 [file Image_2.pdf]

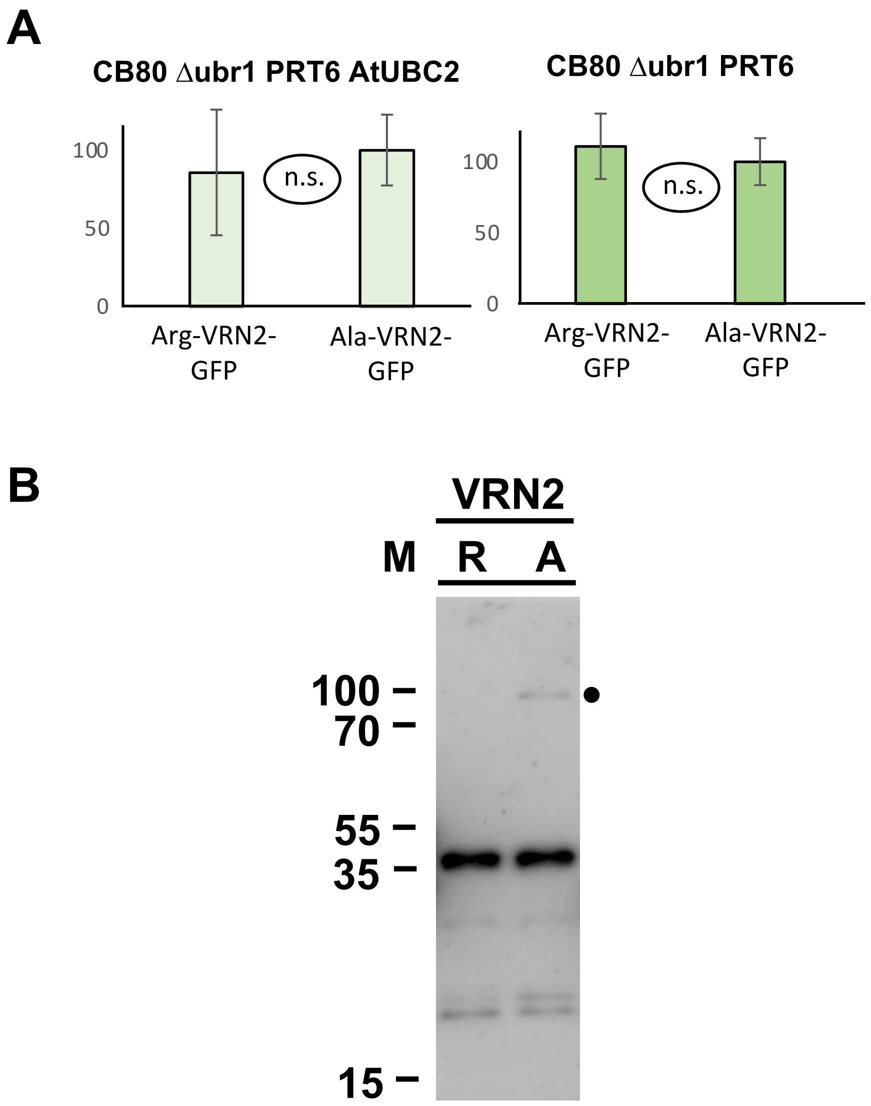

Supplementary Figure 2. Steady-state levels of Arabidopsis Protein VRN2 fused to GFP in different yeast strains. **(A)** Measurement of GFP fluorescence is inconclusive. **(B)** A Western blot experiment indicates a low expression level of the full length protein, but relative enrichment of the version starting with Ala (A) over Arg (R). Dot indicates expected position of full length fusion protein. For abbreviations, see legend to **Figure 2**.
