# Supplementary material for: A Yeast-Based Functional Assay to Study Plant N-Degron – N-Recognin Interactions
Source: Front Plant Sci. 2022 Jan 7;12:806129. doi: 10.3389/fpls.2021.806129 (PMC8777003; doi:10.3389/fpls.2021.806129)
Supplement: Supplementary file 3 [file Data_Sheet_1.pdf]

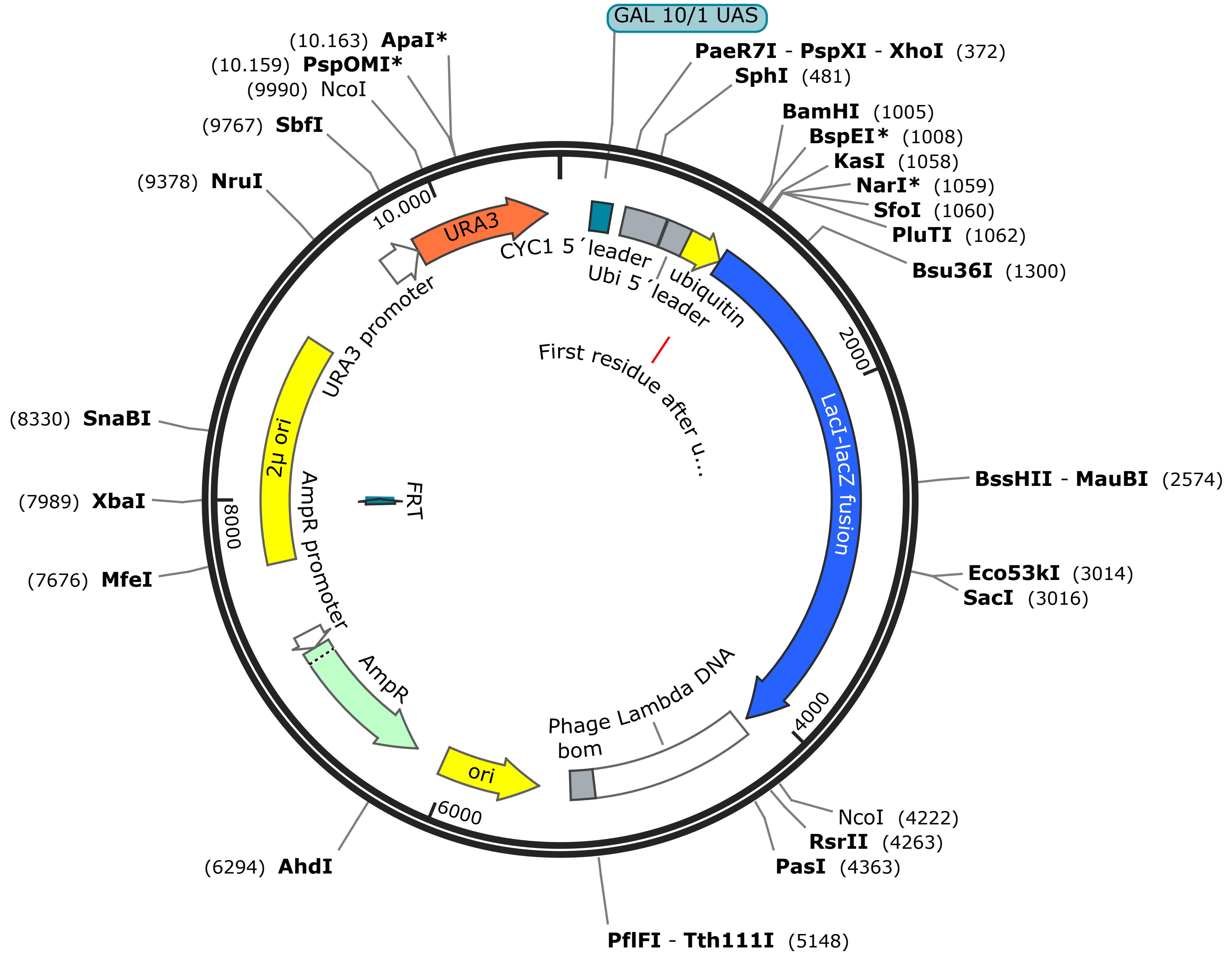

Supplementary File 1 Vector for expression of betaGal substrates
10.666 bp

gatcaaaaatcatcgcttcgctgattaattaccccagaaataaggctaaaaaactaatcgcattatcatcctatggttgttaatttgat
tcgttcatttgaaggtttgtggggccaggttactgccaatttttcctcttcataaccataaaagctagtattgtagaatctttattgttcg
gagcagtgcggcgcgaggcacatctgcgtttcaggaacgcgaccggtgaagacgaggacgcacggaggagagtcttccttcggag
ggctgtcacccgctcggcggcttctaatccgtacttcaatatagcaatgagcagttaagcgtattactgaaagttccaaagagaagg
tttttttaggctaatcgacCTCGAGCAGATCCGCCAGGCGTGTATATAGCGTGGATGGCCAGGCAACTTTAG
TGCTGACACATACAGGCATATATATATGTGTGCGACGACACATGATCATATGGCATGCATGTGCTCT
GTATGTATATAAAACTCTTGTTTTCTTCTTTTCTCTAAATATTCTTTCCTTATACATTAGGTCCTTTGTA
GCATAAATTACTATACTTCTATAGACACGCAAACACAAATACACACACTAAATTACCGGATCAATTCG
CCTGCTTATCTTTCTTCTTCCGAAAGTGCTACTTCAGAAAGAGCAAGAACTGTACGGATAAGGATAA
GTATATCTTCTATCTACTACAATACGAAAAGAACTCTCGAACTCTCCCTCCCACTTTACTTTAACTAAT
AGATTatgcagattttcgtcaagactttgaccggtaaaaccataacattggaagttgaatcttccgataccatcgacaacgttaag
tcgaaaattcaagacaaggaaggtatccctccagatcaacaaagattgatctttgccggtaagcagctagaagacggtagaacgct
gtctgattacaacattcagaaggagtccaccttacatcttgtgctaaggctaagaggtggtatgcacggatccggagcttggctgttg
cccgtctcactggtgaaaagaaaaaccaccctggcgcccaatacgcaaaccgcctctccccgcgcgttggccgattcattaatgcag
ctggcacgacaggtttcccgacttaatcgccttgcagcacatcccccttcgccagctggcgtaatagcgaagaggcccgcaccgat
cgcccttcccaacagttgcgcagcctgaatggcgaatggcgctttgcctggtttccggcaccagaagcggtgccggaaagctggctg
gagtgcgatcttcctgaggccgatactgtcgtcgtcccctcaaactggcagatgcacggttacgatgcgcccatctacaccaacgtg
acctatcccattacggtcaatccgccgtttgttcccacggagaatccgacgggttgttactcgctcacatttaatgttgatgaaagctg
gctacaggaaggccagacgcgaattatttttgatggcgttaactcggcgtttcatctgtggtgcaacgggcgctgggtcggttacggc
caggacagtcgtttgccgtctgaatttgacctgagcgcatttttacgcgccggagaaaaccgcctcgcggtgatggtgctgcgctgga
gtgacggcagttatctggaagatcaggatatgtggcggatgagcggcattttccgtgacgtctcgttgctgcataaaccgactacac
aaatcagcgatttccatgttgccactcgctttaatgatgatttcagccgcgctgtactggaggctgaagttcagatgtgcggcgagtt
gcgtgactacctacgggtaacagtttctttatggcagggtgaaacgcaggtcgccagcggcaccgcgcctttcggcggtgaaattat
cgatgagcgtggtggttatgccgatcgcgtcacactacgtctgaacgtcgaaaacccgaaactgtggagcgccgaaatcccgaatc
tctatcgtgcggtggttgaactgcacaccgccgacggcacgctgattgaagcagaagcctgcgatgtcggtttccgcgaggtgcgga
ttgaaaatggtctgctgctgctgaacggcaagccgttgctgattcgaggcgttaaccgtcacgagcatcatcctctgcatggtcaggt
catggatgagcagacgatggtgcaggatatcctgctgatgaagcagaacaactttaacgccgtgcgctgttcgcattatccgaacca
tccgctgtggtacacgctgtgcgaccgctacggcctgtatgtggtggatgaagccaatattgaaacccacggcatggtgccaatgaa
tcgtctgaccgatgatccgcgctggctaccggcgatgagcgaacgcgtaacgcgaatggtgcagcgcgatcgtaatcacccgagtg
tgatcatctggtcgctggggaatgaatcaggccacggcgctaatcacgacgcgctgtatcgctggatcaaatctgtcgatccttcccg
cccggtgcagtatgaaggcggcggagccgacaccacggccaccgatattatttgcccgatgtacgcgcgcgtggatgaagaccag
cccttcccggctgtgccgaaatggtccatcaaaaaatggctttcgctacctggagagacgcgcccgctgatcctttgcgaatacgccc
acgcgatgggtaacagtcttggcggtttcgctaaatactggcaggcgtttcgtcagtatccccgtttacagggcggcttcgtctggga
ctgggtggatcagtcgctgattaaatatgatgaaaacggcaacccgtggtcggcttacggcggtgattttggcgatacgccgaacga
tcgccagttctgtatgaacggtctggtctttgccgaccgcacgccgcatccagcgctgacggaagcaaaacaccagcagcagttttt
ccagttccgtttatccgggcaaaccatcgaagtgaccagcgaatacctgttccgtcatagcgataacgagctcctgcactggatggt
ggcgctggatggtaagccgctggcaagcggtgaagtgcctctggatgtcgctccacaaggtaaacagttgattgaactgcctgaact
accgcagccggagagcgccgggcaactctggctcacagtacgcgtagtgcaaccgaacgcgaccgcatggtcagaagccgggca
catcagcgcctggcagcagtggcgtctggcggaaaacctcagtgtgacgctccccgccgcgtcccacgccatcccgcatctgaccac
cagcgaaatggatttttgcatcgagctgggtaataagcgttggcaatttaaccgccagtcaggctttctttcacagatgtggattggc
gataaaaaacaactgctgacgccgctgcgcgatcagttcacccgtgcaccgctggataacgacattggcgtaagtgaagcgacccg
cattgaccctaacgcctgggtcgaacgctggaaggcggcgggccattaccaggccgaagcagcgttgttgcagtgcacggcagata
cacttgctgatgcggtgctgattacgaccgctcacgcgtggcagcatcaggggaaaaccttatttatcagccggaaaacctaccgga
ttgatggtagtggtcaaatggcgattaccgttgatgttgaagtggcgagcgatacaccgcatccggcgcggattggcctgaactgcc
agctggcgcaggtagcagagcgggtaaactggctcggattagggccgcaagaaaactatcccgaccgccttactgccgcctgtttt
gaccgctgggatctgccattgtcagacatgtataccccgtacgtcttcccgagcgaaaacggtctgcgctgcgggacgcgcgaattg
aattatggcccacaccagtggcgcggcgacttccagttcaacatcagccgctacagtcaacagcaactgatggaaaccagccatcg

ccatctgctgcacgcggaagaaggcacatggctgaatatcgacggtttccatatggggattggtggcgacgactcctggagcccgtc
agtatcggcggaattccagctgagcgccggtcgctaccattaccagttggtctggtgtcaaaaataataataaccgggcaggccatg
tctgcccgtatttcgcgtaaggaaatccattGTACTGCCGGACCACCGACTGTGAGCCACTCCGGCCATGGCGT
ACGCACTGACCTGCTTACTGATTTGTAAAACCGGTCCGGCCATCACGCTCACATAACGTCCACGCAG
GCTCTCATAGTGAAACGTATCCTCCCCGGTCATCACTGTGCTGCTCTTTTTCGACGCGGCGAACCCCA
GGGAAGCCATCACCCCCACACTGTCCGTCAGCTCATAACGGTACTTCACGTTAATCCCTTTCAGATGA
CTCACACCGGTATCCCCGCCCGACAACGACGGCAATGTACCCGGTTTCACTTGAAAATAGCCCACCG
TAAACGTACCATGTCCACCTTCCGCACGGGCCGGAGTGACTGTCACCGCAAGTGCGGCAAAGACAG
CAACGGCAATACACACATTACGCATCGTTCACCTCTCACTGTTTTATAATAAAACGCCCGTTCCCGGA
CGAACCTCTGTAACACACTCAGACCACGCTGATGCCCAGCGCCTGTTTCTTAATCACCATAACCTGCA
CATCGCTGGCAAACGTATACGGCGGAATATCTGCCGAATGCCGTGTGGACGTAAGCGTGAACGTCA
GGATCACGTTTCCCCGACCCGCTGGCATGTCAACAATACGGGAGAACACCTGTACCGCCTCGTTCGC
CGCGCCATCATAAATCACCGCACCGTTCATCAGTACTTTCAGATAACACATCGAATACGTTGTCCTGC
CGCTGACAGTACGCTTACTTCCGCGAAACGTCAGCGGAAGCACCACTATCTGGCGATCAAAAGGAT
GGTCATCGGTCACGGTGACAGTACGCTGCCTCGCGCGTTTCGGTGATGACGGTGAAAACCTCTGAC
ACATGCAGCTCCCGGAGACGGTCACAGCTTGTCTGTAAGCGGATGCCGGGAGCAGACAAGCCCGTC
AGGGCGCGTCAGCGGGTGTTGGCGGGTGTCGGGGCGCAGCCATGACCCAGTCACGTAGCGATAGC
GGAGTGTATACTGGCTTAACTATGCGGCATCAGAGCAGATTGTACTGAGAGTGCACCATATGCGGT
GTGAAATACCGCACAGATGCGTAAGGAGAAAATACCGCATCAGGCGCTCTTCCGCTTCCTCGCTCAC
TGACTCGCTGCGCTCGGTCGTTCGGCTGCGGCGAGCGGTATCAGCTCACTCAAAGGCGGTAATACG
GTTATCCACAGAATCAGGGGATAACGCAGGAAAGAACATGTGAGCAAAAGGCCAGCAAAAGGCCA
GGAACCGTAAAAAGGCCGCGTTGCTGGCGTTTTTCCATAGGCTCCGCCCCCCTGACGAGCATCACAA
AAATCGACGCTCAAGTCAGAGGTGGCGAAACCCGACAGGACTATAAAGATACCAGGCGTTTCCCCC
TGGAAGCTCCCTCGTGCGCTCTCCTGTTCCGACCCTGCCGCTTACCGGATACCTGTCCGCCTTTCTCCC
TTCGGGAAGCGTGGCGCTTTCTCATAGCTCACGCTGTAGGTATCTCAGTTCGGTGTAGGTCGTTCGC
TCCAAGCTGGGCTGTGTGCACGAACCCCCCGTTCAGCCCGACCGCTGCGCCTTATCCGGTAACTATC
GTCTTGAGTCCAACCCGGTAAGACACGACTTATCGCCACTGGCAGCAGCCACTGGTAACAGGATTAG
CAGAGCGAGGTATGTAGGCGGTGCTACAGAGTTCTTGAAGTGGTGGCCTAACTACGGCTACACTAG
AAGGACAGTATTTGGTATCTGCGCTCTGCTGAAGCCAGTTACCTTCGGAAAAAGAGTTGGTAGCTCT
TGATCCGGCAAACAAACCACCGCTGGTAGCGGTGGTTTTTTTGTTTGCAAGCAGCAGATTACGCGCA
GAAAAAAAGGATCTCAAGAAGATCCTTTGATCTTTTCTACGGGGTCTGACGCTCAGTGGAACGAAAA
CTCACGTTAAGGGATTTTGGTCATGAGATTATCAAAAAGGATCTTCACCTAGATCCTTTTAAATTAAA
AATGAAGTTTTAAATCAATCTAAAGTATATATGAGTAAACTTGGTCTGACAGTTACCAATGCTTAATC
AGTGAGGCACCTATCTCAGCGATCTGTCTATTTCGTTCATCCATAGTTGCCTGACTCCCCGTCGTGTA
GATAACTACGATACGGGAGGGCTTACCATCTGGCCCCAGTGCTGCAATGATACCGCGAGACCCACG
CTCACCGGCTCCAGATTTATCAGCAATAAACCAGCCAGCCGGAAGGGCCGAGCGCAGAAGTGGTCC
TGCAACTTTATCCGCCTCCATCCAGTCTATTAATTGTTGCCGGGAAGCTAGAGTAAGTAGTTCGCCAG
TTAATAGTTTGCGCAACGTTGTTGCCATTGCTGCAGGCATCGTGGTGTCACGCTCGTCGTTTGGTATG
GCTTCATTCAGCTCCGGTTCCCAACGATCAAGGCGAGTTACATGATCCCCCATGTTGTGCAAAAAAG
CGGTTAGCTCCTTCGGTCCTCCGATCGTTGTCAGAAGTAAGTTGGCCGCAGTGTTATCACTCATGGTT
ATGGCAGCACTGCATAATTCTCTTACTGTCATGCCATCCGTAAGATGCTTTTCTGTGACTGGTGAGTA
CTCAACCAAGTCATTCTGAGAATAGTGTATGCGGCGACCGAGTTGCTCTTGCCCGGCGTCAACACGG
GATAATACCGCGCCACATAGCAGAACTTTAAAAGTGCTCATCATTGGAAAACGTTCTTCGGGGCGAA
AACTCTCAAGGATCTTACCGCTGTTGAGATCCAGTTCGATGTAACCCACTCGTGCACCCAACTGATCT
TCAGCATCTTTTACTTTCACCAGCGTTTCTGGGTGAGCAAAAACAGGAAGGCAAAATGCCGCAAAAA
AGGGAATAAGGGCGACACGGAAATGTTGAATACTCATACTCTTCCTTTTTCAATATTATTGAAGCATT
TATCAGGGTTATTGTCTCATGAGCGGATACATATTTGAATGTATTTAGAAAAATAAACAAATAGGGG

TTCCGCGCACATTTCCCCGAAAAGTGCCACCTGACGTCTAAGAAACCATTATTATCATGACATTAACC
TATAAAAATAGGCGTATCACGAGGCCCTTTCGTCTTCAAGAATTCTGAACCAGTCCTAAAACGAGTA
AATAGGACCGGCAATTCTTCAAGCAATAAACAGGAATACCAATTATTAAAAGATAACTTAGTCAGAT
CGTACAATAAAGCTTTGAAGAAAAATGCGCCTTATTCAATCTTTGCTATAAAAAATGGCCCAAAATCT
CACATTGGAAGACATTTGATGACCTCATTTCTTTCAATGAAGGGCCTAACGGAGTTGACTAATGTTGT
GGGAAATTGGAGCGATAAGCGTGCTTCTGCCGTGGCCAGGACAACGTATACTCATCAGATAACAGC
AATACCTGATCACTACTTCGCACTAGTTTCTCGGTACTATGCATATGATCCAATATCAAAGGAAATGA
TAGCATTGAAGGATGAGACTAATCCAATTGAGGAGTGGCAGCATATAGAACAGCTAAAGGGTAGTG
CTGAAGGAAGCATACGATACCCCGCATGGAATGGGATAATATCACAGGAGGTACTAGACTACCTTT
CATCCTACATAAATAGACGCATATAAGTACGCATTTAAGCATAAACACGCACTATGCCGTTCTTCTCA
TGTATATATATATACAGGCAACACGCAGATATAGGTGCGACGTGAACAGTGAGCTGTATGTGCGCA
GCTCGCGTTGCATTTTCGGAAGCGCTCGTTTTCGGAAACGCTTTGAAGTTCCTATTCCGAAGTTCCTA
TTCTCTAGAAAGTATAGGAACTTCAGAGCGCTTTTGAAAACCAAAAGCGCTCTGAAGACGCACTTTC
AAAAAACCAAAAACGCACCGGACTGTAACGAGCTACTAAAATATTGCGAATACCGCTTCCACAAACA
TTGCTCAAAAGTATCTCTTTGCTATATATCTCTGTGCTATATCCCTATATAACCTACCCATCCACCTTTC
GCTCCTTGAACTTGCATCTAAACTCGACCTCTACATTTTTTATGTTTATCTCTAGTATTACTCTTTAGAC
AAAAAAATTGTAGTAAGAACTATTCATAGAGTGAATCGAAAACAATACGAAAATGTAAACATTTCCT
ATACGTAGTATATAGAGACAAAATAGAAGAAACCGTTCATAATTTTCTGACCAATGAAGAATCATCA
ACGCTATCACTTTCTGTTCACAAAGTATGCGCAATCCACATCGGTATAGAATATAATCGGGGATGCCT
TTATCTTGAAAAAATGCACCCGCAGCTTCGCTAGTAATCAGTAAACGCGGGAAGTGGAGTCAGGCTT
TTTTTATGGAAGAGAAAATAGACACCAAAGTAGCCTTCTTCTAACCTTAACGGACCTACAGTGCAAA
AAGTTATCAAGAGACTGCATTATAGAGCGCACAAAGGAGAAAAAAAGTAATCTAAGATGCTTTGTT
AGAAAAATAGCGCTCTCGGGATGCATTTTTGTAGAACAAAAAAGAAGTATAGATTCTTTGTTGGTAA
AATAGCGCTCTCGCGTTGCATTTCTGTTCTGTAAAAATGCAGCTCAGATTCTTTGTTTGAAAAATTAG
CGCTCTCGCGTTGCATTTTTGTTTTACAAAAATGAAGCACAGATTCTTCGTTGGTAAAATAGCGCTTT
CGCGTTGCATTTCTGTTCTGTAAAAATGCAGCTCAGATTCTTTGTTTGAAAAATTAGCGCTCTCGCGT
TGCATTTTTGTTCTACAAAATGAAGCACAGATGCTTCGTTAACAAAGATATGCTATTGAAGTGCAAG
ATGGAAACGCAGAAAATGAACCGGGGATGCGACGTGCAAGATTACCTATGCAATAGATGCAATAGT
TTCTCCAGGAACCGAAATACATACATTGTCTTCCGTAAAGCGCTAGACTATATATTATTATACAGGTT
CAAATATACTATCTGTTTCAGGGAAAACTCCCAGGTTCGGATGTTCAAAATTCAATGATGGGTAACA
AGTACGATCGTAAATCTGTAAAACAGTTTGTCGGATATTAGGCTGTATCTCCTCAAAGCGTATTCGA
ATATCATTGAGAAGCTGCAGCGTCACATCGGATAATAATGATGGCAGCCATTGTAGAAGTGCCTTTT
GCATTTCTAGTCTCTTTCTCGGTCTAGCTAGTTTTACTACATCGCGAAGATAGAATCTTAGATCACACT
GCCTTTGCTGAGCTGGATCAATAGAGTAACAAAAGAGTGGTAAGGCCTCGTTAAAGGACAAGGACC
TGAGCGGAAGTGTATCGTACAGTAGACGGAGTATACTAGTATAGTCTATAGTCCGTGGAATTCTCAT
GTTTGACAGCTTATCATCGATAAGCTTTTCAATTCAATTCATCATTTTTTTTTTATTCTTTTTTTTGATTT
CGGTTTCTTTGAAATTTTTTTGATTCGGTAATCTCCGAACAGAAGGAAGAACGAAGGAAGGAGCACA
GACTTAGATTGGTATATATACGCATATGTAGTGTTGAAGAAACATGAAATTGCCCAGTATTCTTAACC
CAACTGCACAGAACAAAAACCTGCAGGAAACGAAGATAAATCATGTCGAAAGCTACATATAAGGAA
CGTGCTGCTACTCATCCTAGTCCTGTTGCTGCCAAGCTATTTAATATCATGCACGAAAAGCAAACAAA
CTTGTGTGCTTCATTGGATGTTCGTACCACCAAGGAATTACTGGAGTTAGTTGAAGCATTAGGTCCC
AAAATTTGTTTACTAAAAACACATGTGGATATCTTGACTGATTTTTCCATGGAGGGCACAGTTAAGCC
GCTAAAGGCATTATCCGCCAAGTACAATTTTTTACTCTTCGAAGACAGAAAATTTGCTGACATTGGTA
ATACAGTCAAATTGCAGTACTCTGCGGGTGTATACAGAATAGCAGAATGGGCAGACATTACGAATG
CACACGGTGTGGTGGGCCCAGGTATTGTTAGCGGTTTGAAGCAGGCGGCAGAAGAAGTAACAAAG
GAACCTAGAGGCCTTTTGATGTTAGCAGAATTGTCATGCAAGGGCTCCCTATCTACTGGAGAATATA
CTAAGGGTACTGTTGACATTGCGAAGAGCGACAAAGATTTTGTTATCGGCTTTATTGCTCAAAGAGA

CATGGGTGGAAGAGATGAAGGTTACGATTGGTTGATTATGACACCCGGTGTGGGTTTAGATGACAA
GGGAGACGCATTGGGTCAACAGTATAGAACCGTGGATGATGTGGTCTCTACAGGATCTGACATTAT
TATTGTTGGAAGAGGACTATTTGCAAAGGGAAGGGATGCTAAGGTAGAGGGTGAACGTTACAGAA
AAGCAGGCTGGGAAGCATATTTGAGAAGATGCGGCCAGCAAAACTAAAAAACTGTATTATAAGTAA
ATGCATGTATACTAAACTCACAAATTAGAGCTTCAATTTAATTATATCAGTTATTACCC
