# Supplementary material for: A Yeast-Based Functional Assay to Study Plant N-Degron – N-Recognin Interactions
Source: Front Plant Sci. 2022 Jan 7;12:806129. doi: 10.3389/fpls.2021.806129 (PMC8777003; doi:10.3389/fpls.2021.806129)
Supplement: Supplementary file 4 [file Data_Sheet_2.pdf]

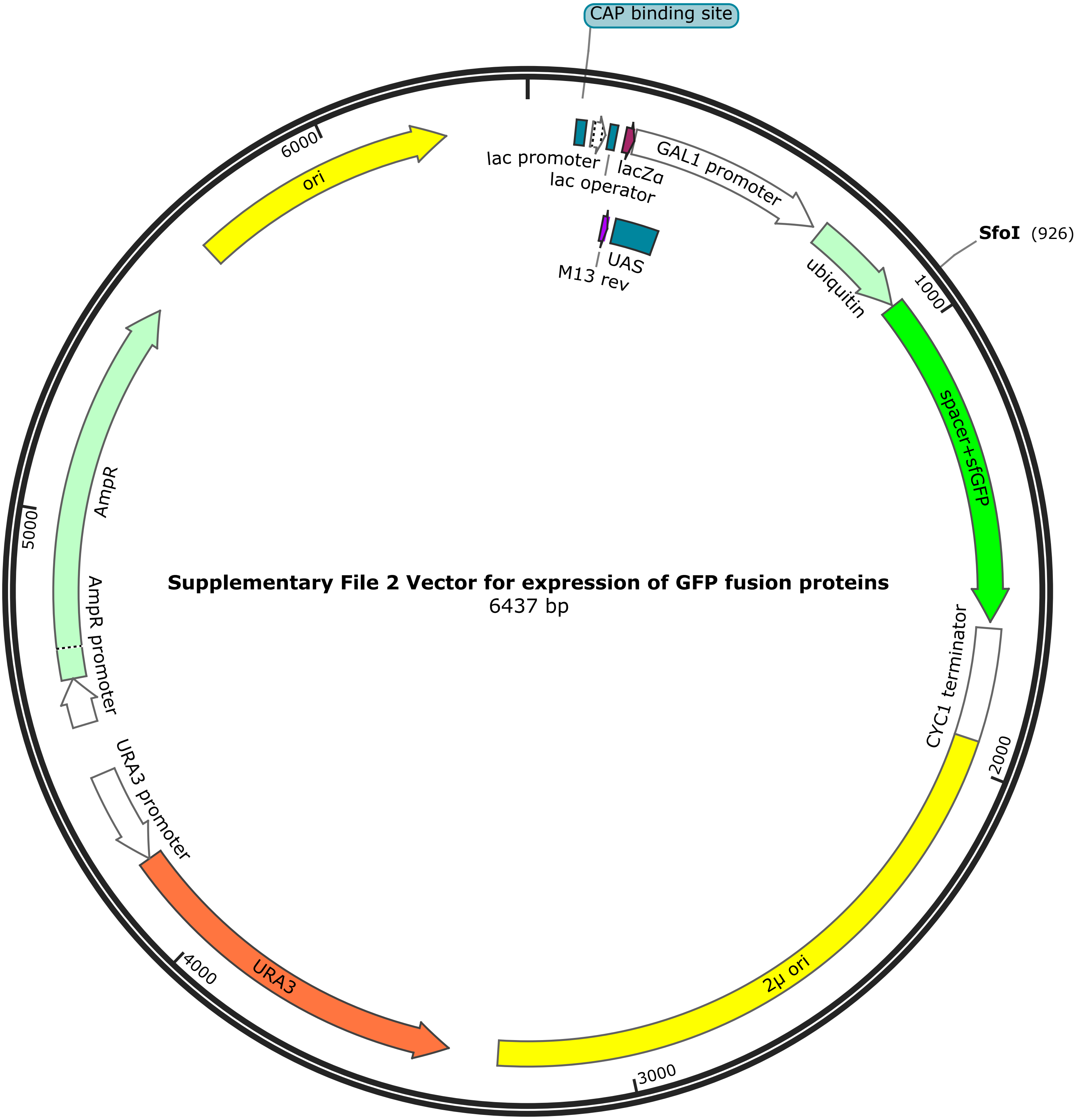

CAP binding site
lac promoter
lac operator
lacZα
GAL1 promoter
M13 rev
UAS
SfoI (926)
1000
ubiquitin
spacer+sfGFP
CYC1 terminator
2000
2μ ori
3000
URA3
4000
URA3 promoter
AmpR promoter
5000
AmpR
6000
ori
Supplementary File 2 Vector for expression of GFP fusion proteins
6437 bp

gcgcccaatacgcaaaccgcctctccccgcgcgttggccgattcattaatgcagctggcacgacaggtttcccgactggaaagcgg
gcagtgagcgcaacgcaattaatgtgagttagctcactcattaggcaccccaggctttacactttatgcttccggctcgtatgttgtgt
ggaattgtgagcggataacaatttcacacaggaaacagctatgaccatgattacgccaagctcggattagaagccgccgagcggg
tgacagccctccgaaggaagactctcctccgtgcgtcctcgtcttcaccggtcgcgttcctgaaacgcagatgtgcctcgcgccgcac
tgctccgaacaataaagattctacaatactagcttttatggttatgaagaggaaaaattggcagtaacctggccccacaaaccttca
aatgaacgaatcaaattaacaaccataggatgataatgcgattagttttttagccttatttctggggtaattaatcagcgaagcgatg
atttttgatctattaacagatatataaatgcaaaaactgcataaccactttaactaatactttcaacattttcggtttgtattacttctta
ttcaaatgtaataaaagtatcaacaaaaaattgttaatatacctctatactttaacgtcaaggagaaaaAACCCCGGATTCTA
GATGCAGATTTTCGTCAAGACTTTGACCGGTAAAACCATAACATTGGAAGTTGAATCTTCCGATACC
ATCGACAACGTTAAGTCGAAAATTCAAGACAAGGAAGGTATCCCTCCAGATCAACAAAGATTGATCT
TTGCCGGTAAGCAGCTAGAAGACGGTAGAACGCTGTCTGATTACAACATTCAGAAGGAGTCCACCTT
ACATCTTGTGCTAAGGCTAAGAGGTGGCGCCGGAGCAGGTGCTGGTGCTGGTGCTGGAGCAATGTC
CAAGGGTGAAGAGCTATTTACTGGGGTTGTACCCATTTTGGTAGAACTGGACGGAGATGTAAACGG
ACATAAATTCTCTGTTAGAGGTGAGGGCGAAGGCGATGCCACCAATGGTAAATTGACTCTGAAGTTT
ATATGCACTACGGGTAAATTACCTGTTCCTTGGCCAACCCTAGTAACAACTTTGACATATGGTGTTCA
ATGTTTCTCAAGATACCCAGACCATATGAAAAGGCATGATTTCTTTAAAAGTGCTATGCCAGAAGGC
TACGTGCAAGAGAGAACTATCTCCTTTAAGGATGACGGTACGTATAAAACACGAGCAGAAGTGAAA
TTCGAAGGGGATACACTAGTTAATCGCATCGAATTAAAGGGTATAGACTTTAAGGAAGATGGTAAT
ATTCTCGGCCATAAACTTGAGTATAATTTCAACTCGCATAATGTGTACATTACAGCTGACAAACAAAA
GAACGGAATTAAAGCGAATTTTAAAATCAGGCACAACGTCGAAGATGGGTCTGTTCAACTTGCCGAT
CATTATCAGCAAAACACCCCTATTGGTGATGGTCCAGTCTTGTTACCCGATAATCACTACTTAAGCAC
ACAGTCTAGATTGTCAAAAGATCCGAATGAAAAGCGTGATCACATGGTTTTATTGGAATTTGTCACC
GCTGCAGGAATAACTCACGGAATGGACGAGCTTTATAAGTAAGGATCCTAGTCGAGTCATGTAATTa
gttatgtcacgcttacattcacgccctccccccacatccgctctaaccgaaaaggaaggagttagacaacctgaagtctaggtccct
atttatttttttatagttatgttagtattaagaacgttatttatatttcaaatttttctttttttctgtacagacgcgtgtacgcatgtaaca
ttatactgaaaaccttgcttgagaaggttttgggacgctcgaaggctttaatttgcaacgaagcatctgtgcttcattttgtagaacaa
aaatgcaacgcgagagcgctaatttttcaaacaaagaatctgagctgcatttttacagaacagaaatgcaacgcgaaagcgctatt
ttaccaacgaagaatctgtgcttcatttttgtaaaacaaaaatgcaacgcgagagcgctaatttttcaaacaaagaatctgagctgc
atttttacagaacagaaatgcaacgcgagagcgctattttaccaacaaagaatctatacttctttttgttctacaaaaatgcatcccg
agagcgctatttttctaacaaagcatcttagattactttttttctcctttgtgcgctctataatgcagtctcttgataactttttgcactgt
aggtccgttaaggttagaagaaggctactttggtgtctattttctcttccataaaaaaagcctgactccacttcccgcgtttactgatta
ctagcgaagctgcgggtgcatttttcaagataaaggcatccccgattatattctataccgatgtggattgcgcatactttgtgaacag
aaagtgatagcgttgatgattcttcattggtcagaaaattatgaacggtttcttctattttgtctctatatactacgtataggaaatgttt
acattttcgtattgttttcgattcactctatgaatagttcttactacaatttttttgtctaaagagtaatactagagataaacataaaaa
atgtagaggtcgagtttagatgcaagttcaaggagcgaaaggtggatgggtaggttatatagggatatagcacagagatatatagc
aaagagatacttttgagcaatgtttgtggaagcggtattcgcaatattttagtagctcgttacagtccggtgcgtttttggtttttgaa
agtgcgtcttcagagcgcttttggttttcaaaagcgctctgaagttcctatactttctagctagagaataggaacttcggaataggaa
cttcaaagcgtttccgaaaacgagcgcttccgaaaatgcaacgcgagctgcgcacatacagctcactgttcacgtcgcacctatatc
tgcgtgttgcctgtatatatatatacatgagaagaacggcatagtgcgtgtttatgcttaaatgcgtacttatatgcgtctatttatgta
ggatgaaaggtagtctagtacctcctgtgatattatcccattccatgcggggtatcgtatgcttccttcagcactaccctttagctgttc
tatatgctgccactcctcaattggattagtctcatccttcaatgctatcatttcctttgatattggatcgatccgatgataagctgtcaaa
catgagaattgggtaataactgatataattaaattgaagctctaatttgtgagtttagtatacatgcatttacttataatacagtttttt
agttttgctggccgcatcttctcaaatatgcttcccagcctgcttttctgtaacgttcaccctctaccttagcatcccttccctttgcaaat
agtcctcttccaacaataataatgtcagatcctgtagagaccacatcatccacggttctatactgttgacccaatgcgtctcccttgtc
atctaaacccacaccgggtgtcataatcaaccaatcgtaaccttcatctcttccacccatgtctctttgagcaataaagccgataaca
aaatctttgtcgctcttcgcaatgtcaacagtacccttagtatattctccagtagatagggagcccttgcatgacaattctgctaacat
caaaaggcctctaggttcctttgttacttcttctgccgcctgcttcaaaccgctaacaatacctgggcccaccacaccgtgtgcattcg

taatgtctgcccattctgctattctgtatacacccgcagagtactgcaatttgactgtattaccaatgtcagcaaattttctgtcttcga
agagtaaaaaattgtacttggcggataatgcctttagcggcttaactgtgccctccatggaaaaatcagtcaagatatccacatgtgt
ttttagtaaacaaattttgggacctaatgcttcaactaactccagtaattccttggtggtacgaacatccaatgaagcacacaagtttg
tttgcttttcgtgcatgatattaaatagcttggcagcaacaggactaggatgagtagcagcacgttccttatatgtagctttcgacatg
atttatcttcgtttcctgcatgtttttgttctgtgcagttgggttaagaatactgggcaatttcatgtttcttcaacactacatatgcgtat
atataccaatctaagtctgtgctccttccttcgttcttccttctgttcggagattaccgaatcaaaaaaatttcaaagaaaccgaaatc
aaaaaaaagaataaaaaaaaaaatgatgaattgaattgaaaagctaattcttgaagacgaaagggcctcgtgatacgcctatttttta
taggttaatgtcatgataataatggtttcttagacgtcaggtggcacttttcggggaaatgtgcgcggaacccctatttgtttatttttct
aaatacattcaaatatgtatccgctcatgagacaataaccctgataaatgcttcaataatattgaaaaaggaagagtatgagtattc
aacatttccgtgtcgcccttattcccttttttgcggcattttgccttcctgtttttgctcacccagaaacgctggtgaaagtaaaagatgc
tgaagatcagttgggtgcacgagtgggttacatcgaactggatctcaacagcggtaagatccttgagagttttcgccccgaagaacg
ttttccaatgatgagcacttttaaagttctgctatgtggcgcggtattatcccgtattgacgccgggcaagagcaactcggtcgccgca
tacactattctcagaatgacttggttgagtactcaccagtcacagaaaagcatcttacggatggcatgacagtaagagaattatgca
gtgctgccataaccatgagtgataacactgcggccaacttacttctgacaacgatcggaggaccgaaggagctaaccgcttttttgc
acaacatgggggatcatgtaactcgccttgatcgttgggaaccggagctgaatgaagccataccaaacgacgagcgtgacaccac
gatgcctgtagcaatggcaacaacgttgcgcaaactattaactggcgaactacttactctagcttcccggcaacaattaatagactg
gatggaggcggataaagttgcaggaccacttctgcgctcggcccttccggctggctggtttattgctgataaatctggagccggtga
gcgtgggtctcgcggtatcattgcagcactggggccagatggtaagccctcccgtatcgtagttatctacacgacggggagtcaggc
aactatggatgaacgaaatagacagatcgctgagataggtgcctcactgattaagcattggtaactgtcagaccaagtttactcata
tatactttagattgatttaaaacttcatttttaatttaaaaggatctaggtgaagatcctttttgataatctcatgaccaaaatcccttaa
cgtgagttttcgttccactgagcgtcagaccccgtagaaaagatcaaaggatcttcttgagatcctttttttctgcgcgtaatctgctgc
ttgcaaacaaaaaaaccaccgctaccagcggtggtttgtttgccggatcaagagctaccaactctttttccgaaggtaactggcttca
gcagagcgcagataccaaatactgtccttctagtgtagccgtagttaggccaccacttcaagaactctgtagcaccgcctacatacc
tcgctctgctaatcctgttaccagtggctgctgccagtggcgataagtcgtgtcttaccgggttggactcaagacgatagttaccggat
aaggcgcagcggtcgggctgaacggggggttcgtgcacacagcccagcttggagcgaacgacctacaccgaactgagatacctac
agcgtgagctatgagaaagcgccacgcttcccgaagggagaaaggcggacaggtatccggtaagcggcagggtcggaacagga
gagcgcacgagggagcttccagggggaaacgcctggtatctttatagtcctgtcgggtttcgccacctctgacttgagcgtcgatttt
tgtgatgctcgtcaggggggcggagcctatggaaaaacgccagcaacgcggcctttttacggttcctggccttttgctggccttttgctc
acatgttctttcctgcgttatcccctgattctgtggataaccgtattaccgcctttgagtgagctgataccgctcgccgcagccgaacg
accgagcgcagcgagtcagtgagcgaggaagcggaaga
