# Supplementary material for: A Yeast-Based Functional Assay to Study Plant N-Degron – N-Recognin Interactions
Source: Front Plant Sci. 2022 Jan 7;12:806129. doi: 10.3389/fpls.2021.806129 (PMC8777003; doi:10.3389/fpls.2021.806129)
Supplement: Supplementary file 5 [file Data_Sheet_3.pdf]

Supplementary File 3.
DNA insertions in vector for expression of GFP fusions (Sfo I site; see **Supplementary File 2**):

V-GFP:
GTTCATTCTGGTGGTGGTGGCGGTGGTAGAGGTGGATCCGGAGCTTGGCTGTTGCCCGTCTCACTG
GTGAAAAGAAAAACCACCCTGGCGCCCAATACGCAAACCGCCTCTCCCCGCGCGTTGGCCGATTCAT
TAATGCAGCATATGGGATCC

R-GFP:
AGACATTCTGGTGGTGGTGGCGGTGGTAGAGGTGGATCCGGAGCTTGGCTGTTGCCCGTCTCACTG
GTGAAAAGAAAAACCACCCTGGCGCCCAATACGCAAACCGCCTCTCCCCGCGCGTTGGCCGATTCAT
TAATGCAGCATATGGGATCC

RD-HRE2-GFP:
AGAGATGGGGGAGCTATCATTTCTGATTTCATCTGGTCGAAATCTGAGTCAGAACCGAGTCAACTCG
GCTCTGTTAGCAGCAGGAAGAAGCGTAAACCCGTCTCAGTGAGTGAAGAAAGAGATGGGAAACGA
GAGAGGAAGAATCTGTACAGAGGGATAAGGCAGAGGCCATGGGGCAAATGGGCAGCGGAGATTC
GTGACCCGAGCAAAGGTGTACGTGTCTGGCTTGGCACATTCAAAACCGCCGACGAAGCTGCTCGAG
CCTACGACGTTGCTGCCATCAAAATCCGTGGCCGGAAAGCCAAACTGAATTTCCCAAACACTCAAGT
AGAAGAAGAAGCCGATACTAAACCAGGGGGGAATCAAAATGAGCTGATTTCGGAAAACCAAGTAG
AGAGCTTATCGGAGGACCTGATGGCATTGGAGGATTACATGAGATTCTATCAGATTCCGGTTGCCG
ACGACCAATCGGCGACCGATATTGGAAATTTATGGAGCTATCAAGACTCCAAT

AD-HRE2-GFP:
GCTGATGGGGGAGCTATCATTTCTGATTTCATCTGGTCGAAATCTGAGTCAGAACCGAGTCAACTCG
GCTCTGTTAGCAGCAGGAAGAAGCGTAAACCCGTCTCAGTGAGTGAAGAAAGAGATGGGAAACGA
GAGAGGAAGAATCTGTACAGAGGGATAAGGCAGAGGCCATGGGGCAAATGGGCAGCGGAGATTC
GTGACCCGAGCAAAGGTGTACGTGTCTGGCTTGGCACATTCAAAACCGCCGACGAAGCTGCTCGAG
CCTACGACGTTGCTGCCATCAAAATCCGTGGCCGGAAAGCCAAACTGAATTTCCCAAACACTCAAGT
AGAAGAAGAAGCCGATACTAAACCAGGGGGGAATCAAAATGAGCTGATTTCGGAAAACCAAGTAG
AGAGCTTATCGGAGGACCTGATGGCATTGGAGGATTACATGAGATTCTATCAGATTCCGGTTGCCG
ACGACCAATCGGCGACCGATATTGGAAATTTATGGAGCTATCAAGACTCCAAT

RD-VRN2-GFP:
AGAGATAGGCAGAATTGTCGCGCGAAATCCTCACCGGAGGAAGTGATTTCAACTGATGAGAATCTC
TTGATATATTGTAAACCTGTTCGACTATATAACATCTTTCACCTTCGCTCTCTAGGCAACCCATCGTTT
CTGCCAAGATGCTTGAACTACAAAATTGGGGCAAAGCGCAAAAGAAAGTCAAGATCTACTGGGATG
GTAGTTTTCAACTATAAGGATTGTAATAATACATTACAAAGAACTGAAGTTAGGGAGGATTGTTCTT
GTCCATTTTGCTCTATGCTATGTGGTAGCTTCAAGGGGCTGCAATTTCATTTGAATTCATCTCATGATT
TATTTGAATTTGAGTTCAAGCTTTTGGAAGAATACCAGACAGTTAATGTTTCTGTAAAACTTAATTCC
TTCATATTTGAGGAAGAAGGAAGTGATGATGATAAATTTGAGCCCTTCTCTCTCTGCTCGAAACCTC
GTAAGCGTAGACAAAGAGGTGGCAGAAATAACACCAGGAGACTTAAAGTATGCTTTTTACCGTTGG
ATTCACCCAGTTTAGCTAATGGCACAGAAAATGGAATTGCCCTGCTGAATGATGGAAACCGTGGTTT
AGGATATCCCGAGGCAACAGAGCTTGCTGGACAATTTGAGATGACTAGCAACATTCCACCAGCCATA
GCCCACTCTTCTCTGGACGCTGGTGCTAAAGTTATATTAACAACCGAAGCTGTGGTCCCTGCTACTAA
GACAAGAAAGTTATCTGCTGAGCGATCAGAGGCTAGAAGCCACCTACTTCTTCAGAAACGCCAATTC
TATCATTCTCACAGAGTCCAGCCAATGGCGCTTGAGCAAGTAATGTCTGATCGGGATAGCGAGGAT

GAAGTCGATGACGATGTTGCAGATTTTGAAGATCGCCAGATGCTTGATGACTTTGTGGATGTGAATA
AAGATGAAAAGCAATTCATGCATCTTTGGAACTCGTTTGTAAGAAAACAAAGGGTTATAGCAGATG
GTCATATCTCTTGGGCATGTGAAGTATTTTCAAGATTTTACGAGAAAGAGTTGCACTGTTACTCATCA
CTCTTCTGGTGTTGGAGATTGTTTTTGATTAAACTATGGAACCATGGACTTGTCGACTCAGCCACCAT
CAACAACTGCAATACCATCCTCGAGAATTGCCGTAATACCTCAGTCACTAACAACAACAACAACAGT
GTGGATCATCCCAGTGACTCAAACACCAACAACAATAACATTGTGGATCATCCGAATGACATAAAAA
ACAAGAACAATGTTGACAACAAGGACAATAACAGCAGAGACAAG

AD-VRN2-GFP
GCTGATAGGCAGAATTGTCGCGCGAAATCCTCACCGGAGGAAGTGATTTCAACTGATGAGAATCTC
TTGATATATTGTAAACCTGTTCGACTATATAACATCTTTCACCTTCGCTCTCTAGGCAACCCATCGTTT
CTGCCAAGATGCTTGAACTACAAAATTGGGGCAAAGCGCAAAAGAAAGTCAAGATCTACTGGGATG
GTAGTTTTCAACTATAAGGATTGTAATAATACATTACAAAGAACTGAAGTTAGGGAGGATTGTTCTT
GTCCATTTTGCTCTATGCTATGTGGTAGCTTCAAGGGGCTGCAATTTCATTTGAATTCATCTCATGATT
TATTTGAATTTGAGTTCAAGCTTTTGGAAGAATACCAGACAGTTAATGTTTCTGTAAAACTTAATTCC
TTCATATTTGAGGAAGAAGGAAGTGATGATGATAAATTTGAGCCCTTCTCTCTCTGCTCGAAACCTC
GTAAGCGTAGACAAAGAGGTGGCAGAAATAACACCAGGAGACTTAAAGTATGCTTTTTACCGTTGG
ATTCACCCAGTTTAGCTAATGGCACAGAAAATGGAATTGCCCTGCTGAATGATGGAAACCGTGGTTT
AGGATATCCCGAGGCAACAGAGCTTGCTGGACAATTTGAGATGACTAGCAACATTCCACCAGCCATA
GCCCACTCTTCTCTGGACGCTGGTGCTAAAGTTATATTAACAACCGAAGCTGTGGTCCCTGCTACTAA
GACAAGAAAGTTATCTGCTGAGCGATCAGAGGCTAGAAGCCACCTACTTCTTCAGAAACGCCAATTC
TATCATTCTCACAGAGTCCAGCCAATGGCGCTTGAGCAAGTAATGTCTGATCGGGATAGCGAGGAT
GAAGTCGATGACGATGTTGCAGATTTTGAAGATCGCCAGATGCTTGATGACTTTGTGGATGTGAATA
AAGATGAAAAGCAATTCATGCATCTTTGGAACTCGTTTGTAAGAAAACAAAGGGTTATAGCAGATG
GTCATATCTCTTGGGCATGTGAAGTATTTTCAAGATTTTACGAGAAAGAGTTGCACTGTTACTCATCA
CTCTTCTGGTGTTGGAGATTGTTTTTGATTAAACTATGGAACCATGGACTTGTCGACTCAGCCACCAT
CAACAACTGCAATACCATCCTCGAGAATTGCCGTAATACCTCAGTCACTAACAACAACAACAACAGT
GTGGATCATCCCAGTGACTCAAACACCAACAACAATAACATTGTGGATCATCCGAATGACATAAAAA
ACAAGAACAATGTTGACAACAAGGACAATAACAGCAGAGACAAG

RD-ZPR2-GFP
GATCTGACAACTTCAGAGCCACCATTCCCAGACACTGACACACCAACTATGAGATCTGCTTCGTACCA
TATCAAACATAAATCTAAGACACAAACCCATCTTCGTATCCTTAATCTCACCAGCAGGAGGAGAAGA
TTACTGAAGGAACAGAAGGAGATGGAGATGAGGAACTTGAAGCTTTTCGTAGAGAATCAAAGCATC
ATACGAGAGAATGAAGCTTTAAAAAAGAAAGCTCTTCTTCTCCACCATGAAAACAATGCTCTGTTTG
CTCTGCTTCACCCAAAATACTCCCCTGTTTCAACCTCCTTGCTTCAG

AD-ZPR2-GFP
GCTGATCTGACAACTTCAGAGCCACCATTCCCAGACACTGACACACCAACTATGAGATCTGCTTCGTA
CCATATCAAACATAAATCTAAGACACAAACCCATCTTCGTATCCTTAATCTCACCAGCAGGAGGAGA
AGATTACTGAAGGAACAGAAGGAGATGGAGATGAGGAACTTGAAGCTTTTCGTAGAGAATCAAAG
CATCATACGAGAGAATGAAGCTTTAAAAAAGAAAGCTCTTCTTCTCCACCATGAAAACAATGCTCTG
TTTGCTCTGCTTCACCCAAAATACTCCCCTGTTTCAACCTCCTTGCTTCAG

RD-BBX30-GFP
AGAGATAGAGGGTTTGAGAAAGAAGAAGAGAGAAGAAGCGACAATGGAGGATGCCAAAGACTAT
GCACGGAGAGTCACAAAGCTCCGGTAAGCTGTGAGCTTTGCGGCGAGAACGCCACCGTGTATTGTG

AGGCAGACGCAGCTTTCCTTTGTAGGAAATGCGATCGATGGGTCCATTCTGCTAATTTTCTAGCTCG
GAGACATCTCCGGCGCGTGATCTGCACGACCTGTCGGAAGCTAACTCGTCGATGTCTTGTCGGTGAT
AATTTTAATGTTGTTTTACCGGAGATAAGGATGATAGCAAGGATTGAAGAACATAGTAGTGATCACA
AAATTCCCTTTGTGTTTCTC

AD-BBX30-GFP
GCTGATAGAGGGTTTGAGAAAGAAGAAGAGAGAAGAAGCGACAATGGAGGATGCCAAAGACTAT
GCACGGAGAGTCACAAAGCTCCGGTAAGCTGTGAGCTTTGCGGCGAGAACGCCACCGTGTATTGTG
AGGCAGACGCAGCTTTCCTTTGTAGGAAATGCGATCGATGGGTCCATTCTGCTAATTTTCTAGCTCG
GAGACATCTCCGGCGCGTGATCTGCACGACCTGTCGGAAGCTAACTCGTCGATGTCTTGTCGGTGAT
AATTTTAATGTTGTTTTACCGGAGATAAGGATGATAGCAAGGATTGAAGAACATAGTAGTGATCACA
AAATTCCCTTTGTGTTTCTC

RD-BBX31-GFP
GCTGATAGAGGCTTGAATAATGAAGAGAGCAGAAGAAGTGACGGAGGAGGTTGCCGGAGTCTCTG
CACGAGACCGAGTGTTCCGGTAAGGTGTGAGCTTTGCGACGGAGACGCCTCCGTGTTCTGTGAAGC
GGACTCGGCGTTCCTCTGTAGAAAATGTGACCGGTGGGTTCATGGAGCGAATTTTCTAGCTTGGAG
ACACGTAAGGCGCGTGCTATGCACTTCTTGTCAGAAACTCACGCGCCGGTGCCTCGTCGGAGATCAT
GACTTCCACGTTGTTTTACCGTCGGTGACGACGGTCGGAGAAACCACCGTGGAGAATAGAAGTGAA
CAAGATAATCATGAGGTTCCGTTTGTTTTTCTC

AD-BBX31-GFP
GCTGATAGAGGCTTGAATAATGAAGAGAGCAGAAGAAGTGACGGAGGAGGTTGCCGGAGTCTCTG
CACGAGACCGAGTGTTCCGGTAAGGTGTGAGCTTTGCGACGGAGACGCCTCCGTGTTCTGTGAAGC
GGACTCGGCGTTCCTCTGTAGAAAATGTGACCGGTGGGTTCATGGAGCGAATTTTCTAGCTTGGAG
ACACGTAAGGCGCGTGCTATGCACTTCTTGTCAGAAACTCACGCGCCGGTGCCTCGTCGGAGATCAT
GACTTCCACGTTGTTTTACCGTCGGTGACGACGGTCGGAGAAACCACCGTGGAGAATAGAAGTGAA
CAAGATAATCATGAGGTTCCGTTTGTTTTTCTC

RD-bHLH38-GFP
AGAGATGCATTAGTCCCTTCATTTTTCACAAACTTCGGTTGGCCGTCAACGAATCAATACGAAAGCTA
TTACGGTGCCGGAGATAACCTAAATAACGGCACATTTCTTGAATTGACGGTACCACAGACTTATGAA
GTGACTCATCATCAGAATAGCTTGGGAGTATCTGTTTCGTCAGAAGGAAATGAGATAGACAACAATC
CGGTTGTGGTCAAGAAGCTTAATCACAATGCTAGTGAACGTGACCGACGCAAGAAGATCAACACTTT
GTTCTCATCTCTCCGTTCATGTCTTCCAGCTTCTGATCAATCGAAGAAGCTAAGTATTCCTGAAACGG
TTTCAAAGAGCTTAAAGTACATACCAGAGCTGCAACAGCAAGTGAAGAGGCTAATACAAAAGAAGG
AAGAAATTTTGGTACGAGTATCGGGTCAAAGAGACTTTGAGCTTTACGATAAGCAGCAACCAAAGG
CGGTCGCGAGTTATCTCTCAACGGTTTCTGCCACTAGGCTTGGTGACAACGAAGTGATGGTCCAAGT
CTCATCGTCCAAGATTCATAACTTTTCGATATCAAATGTGTTGGGTGGGATAGAAGAAGATGGGTTT
GTTCTTGTGGATGTTTCATCATCAAGATCTCAAGGAGAGAGGCTCTTCTACACTTTGCATCTTCAAGT
GGAGAATATGGATGATTACAAGATTAATTGCGAAGAATTAAGTGAAAGGATGTTGTACTTGTACGA
GAAATGTGAAAACTCGTTTAAC

AD-bHLH38-GFP
GCTGATGCATTAGTCCCTTCATTTTTCACAAACTTCGGTTGGCCGTCAACGAATCAATACGAAAGCTA
TTACGGTGCCGGAGATAACCTAAATAACGGCACATTTCTTGAATTGACGGTACCACAGACTTATGAA
GTGACTCATCATCAGAATAGCTTGGGAGTATCTGTTTCGTCAGAAGGAAATGAGATAGACAACAATC

CGGTTGTGGTCAAGAAGCTTAATCACAATGCTAGTGAACGTGACCGACGCAAGAAGATCAACACTTT
GTTCTCATCTCTCCGTTCATGTCTTCCAGCTTCTGATCAATCGAAGAAGCTAAGTATTCCTGAAACGG
TTTCAAAGAGCTTAAAGTACATACCAGAGCTGCAACAGCAAGTGAAGAGGCTAATACAAAAGAAGG
AAGAAATTTTGGTACGAGTATCGGGTCAAAGAGACTTTGAGCTTTACGATAAGCAGCAACCAAAGG
CGGTCGCGAGTTATCTCTCAACGGTTTCTGCCACTAGGCTTGGTGACAACGAAGTGATGGTCCAAGT
CTCATCGTCCAAGATTCATAACTTTTCGATATCAAATGTGTTGGGTGGGATAGAAGAAGATGGGTTT
GTTCTTGTGGATGTTTCATCATCAAGATCTCAAGGAGAGAGGCTCTTCTACACTTTGCATCTTCAAGT
GGAGAATATGGATGATTACAAGATTAATTGCGAAGAATTAAGTGAAAGGATGTTGTACTTGTACGA
GAAATGTGAAAACTCGTTTAAC

RD-RIN4-II-GFP
AGAGATTGGGAAGCTGAGGAGAATGTTCCTTACACAGCTTACTTTGACAAAGCTCGTAAGACTCGA
GCACCCGGTAGCAAGATCATGAACCCGAATGACCCGGAGTATAACTCTGACTCTCAATCACAAGCTC
CTCCTCATCCTCCTTCTTCCAGAACCAAACCTGAGCAAGTTGACACGGTTAGAAGATCACGTGAGCAT
ATGAGAAGCCGAGAAGAGAGCGAGTTGAAACAGTTTGGTGATGCTGGTGGTTCATCAAATGAAGCT
GCTAACAAAAGACAAGGAAGAGCTTCTCAGAACAATAGTTATGACAACAAGTCACCTTTGCATAAG
AATTCTTATGATGGTACTGGAAAATCTAGGCCTAAACCTACCAACCTTAGAGCTGATGAAAGTCCTG
AAAAAGTCACAGTGGTGCCTAAATTCGGT

AD-RIN4-II-GFP
GCTGATTGGGAAGCTGAGGAGAATGTTCCTTACACAGCTTACTTTGACAAAGCTCGTAAGACTCGAG
CACCCGGTAGCAAGATCATGAACCCGAATGACCCGGAGTATAACTCTGACTCTCAATCACAAGCTCC
TCCTCATCCTCCTTCTTCCAGAACCAAACCTGAGCAAGTTGACACGGTTAGAAGATCACGTGAGCATA
TGAGAAGCCGAGAAGAGAGCGAGTTGAAACAGTTTGGTGATGCTGGTGGTTCATCAAATGAAGCT
GCTAACAAAAGACAAGGAAGAGCTTCTCAGAACAATAGTTATGACAACAAGTCACCTTTGCATAAG
AATTCTTATGATGGTACTGGAAAATCTAGGCCTAAACCTACCAACCTTAGAGCTGATGAAAGTCCTG
AAAAAGTCACAGTGGTGCCTAAATTCGGT

RD-RIN4-III-GFP
AGAGACTGGGACGAGAACAACCCGTCATCAGCTGACGGATACACGCATATCTTCAATAAAGTCCGT
GAAGAGAGAAGTTCTGGAGCAAATGTGAGTGGATCTTCAAGAACGCCGACTCATCAAAGCTCCCGT
AACCCGAACAACACTTCCTCATGTTGCTGCTTTGGCTTTGGAGGAAAA

AD-RIN4-III-GFP
GCTGACTGGGACGAGAACAACCCGTCATCAGCTGACGGATACACGCATATCTTCAATAAAGTCCGTG
AAGAGAGAAGTTCTGGAGCAAATGTGAGTGGATCTTCAAGAACGCCGACTCATCAAAGCTCCCGTA
ACCCGAACAACACTTCCTCATGTTGCTGCTTTGGCTTTGGAGGAAAA
