# Supplementary material for: A Yeast-Based Functional Assay to Study Plant N-Degron – N-Recognin Interactions
Source: Front Plant Sci. 2022 Jan 7;12:806129. doi: 10.3389/fpls.2021.806129 (PMC8777003; doi:10.3389/fpls.2021.806129)
Supplement: Supplementary file 6 [file Data_Sheet_4.pdf]

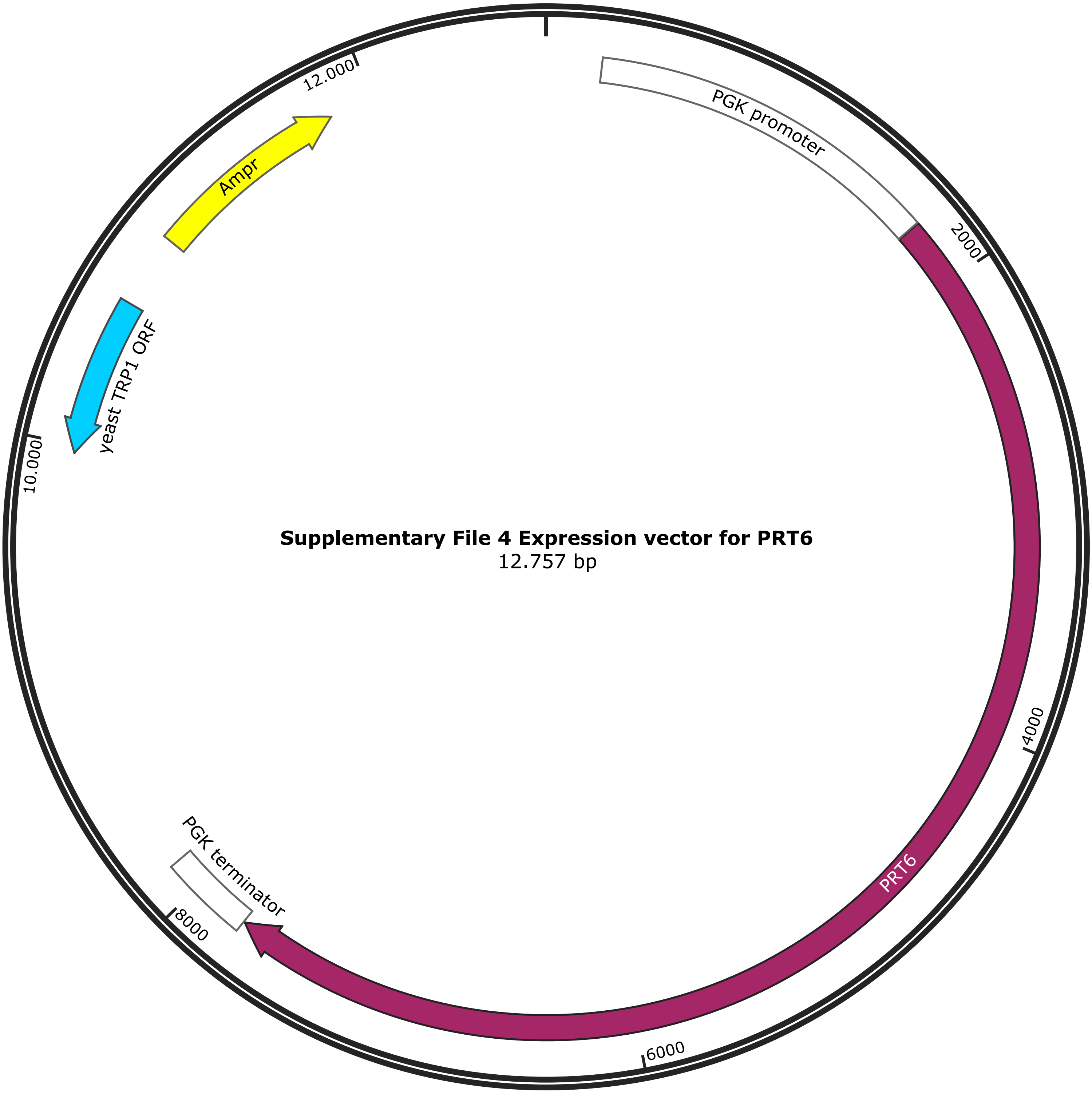
Supplementary File 4 Expression vector for PRT6
12.757 bp
PGK promoter
PRT6
PGK terminator
Ampr
yeast TRP1 ORF
2000
4000
6000
8000
10.000
12.000

gcgcccaatacgcaaaccgcctctccccgcgcgttggccgattcattaatgcagctggcacgacaggtttcccgactggaaagcgg
gcagtgagcgcaacgcaattaatgtgagttagctcactcattaggcaccccaggctttacactttatgcttccggctcgtatgttgtgt
ggaattgtgagcggataacaatttcacacaggaaacagctatgaccatgattacgccaagctttctaactgatctatccaaaactga
aaattacattcttgattaggtttatcacaggcaaatgtaatttgtggtattttgccgttcaaaatctgtagaattttctcattggtcacat
tacaacctgaaaatactttatctacaatcataccattcttataacatgtccccttaatactaggatcaggcatgaacgcatcacagac
aaaatcttcttgacaaacgtcacaattgatccctccccatccgttatcacaatgacaggtgtcattttgtgctcttatgggacgatcctt
attaccgctttcatccggtgatagaccgccacagaggggcagagagcaatcatcacctgcaaacccttctatacactcacatctacc
agtgtacgaattgcattcagaaaactgtttgcattcaaaaataggtagcatacaattaaaacatggcgggcatgtatcattgccctta
tcttgtgcagttagacgcgaatttttcgaagaagtaccttcaaagaatggggtcttatcttgttttgcaagtaccactgagcaggataa
taatagaaatgataatatactatagtagagataacgtcgatgacttcccatactgtaattgcttttagttgtgtatttttagtgtgcaag
tttctgtaaatcgattaattttttttctttcctctttttattaaccttaatttttattttagattcctgacttcaactcaagacgcacagata
ttataacatctgcataataggcatttgcaagaattactcgtgagtaaggaaagagtgaggaactatcgcatacctgcatttaaagat
gccgatttgggcgcgaatcctttattttggcttcaccctcatactattatcagggccagaaaaaggaagtgtttccctccttcttgaatt
gatgttaccctcataaagcacgtggcctcttatcgagaaagaaattaccgtcgctcgtgatttgtttgcaaaaagaacaaaactgaa
aaaacccagacacgctcgacttcctgtcttcctattgattgcagcttccaatttcgtcacacaacaaggtcctagcgacggctcacag
gttttgtaacaagcaatcgaaggttctggaatggcgggaaagggtttagtaccacatgctatgatgcccactgtgatctccagagca
aagttcgttcgatcgtactgttactctctctttcaaacagaattgtccgaatcgtgtgacaacaacagcctgttctcacacactcttt
tcttctaaccaagggggtggtttagtttagtagaacctcgtgaaacttacatttacatatatataaacttgcataaattggtcaatgca
agaaatacatatttggtcttttctaattcgtagtttttcaagttcttagatgctttctttttctctttttttacagatcatcaaggaagtaatt
atctacttttacaacaaatataaaaccaaaagatcccataCCATGGAGACCAACTCTTCTCTTTTTGGTTTAGTTTC
TCCTAGCTCGCATGATTTGGTCATTGAGAGGCTTGCTTCTGTGGGGGTTCCTAAGAAATACCGCTCTA
AACGTGGCCTAGTGGAATTCGTTAGAGCTAACCCGGCCAAGATTTCAGAGTTAGTCTCTGCTCTCTT
ACCTACAGATGACGATGTTAAACTAGGATTGAAAGAAGCTAGGGAACGGCCTCGAAAGTCTGCTGT
GAGTCCTACCATGAAAAAGAGGTTCAGGGAGAGCATGAATATGCTGCAGTGGTTGATGTTCCAGGA
TGAGCCTGATGTTTCTTTGAGGAATCTAGCAAAACTGAATCTTGATCAGCGAGGTGTTTGTGGCTCT
GTCTGGGGACAGAATGATATAGCATATAGGTGTAGGACATGCGAGAATGACCCAACTTGTGCAATC
TGCGTGCCTTGTTTCCAGAATGGGGATCACAACTCCCACGATTATTCAATCATCTACACAGGTGGTG
GTTGTTGTGATTGTGGGGATGAAACAGCATGGAAACCTGATGGTTTCTGTTCAAATCATAAAGGTTC
TGAACAGATTCGACCCCTCTCAGAAAATCTAGCAAATTCAGTTGGGCCTATACTTGATGCCCTTTTTA
CTTGTTGGAATAACAAGCTCTTATCTGCAGAAAGTAGTGGTCAGAAAGGTGCTAGATCCAACGATAC
TCTTGTGATACTCCAAAAGATGTCAAACGAGCTGACATTTATAGTGGTTGAAATGCTTCTGGAGTTTT
CTATGTCCAGTGAGAGTTTGCTCAGTTTTGTTTCTAGAAGGATTATCTCTTCAAGTGGTTTATTGAGC
ATTCTCTTAAAGGCTGAGAGGTTCTTGGACCAAGATGTTATGAAGAAACTACATGACTTGTTCCTCA
AATTAATAGGAGATCCAGTCTTCAAGTGTGAATTTGCTAAAGCATTTGTGAGTTATTATCCAGTTGTG
ATAAGCGAAGTGGTTAAGCAGGGTACTGATAATGCATTCAAGAAATATCCTCTACTGTCCACATTTT
CTGTGCAAATCCTCACGGTGCCAACTCTAACACCATTTCTGGTGAAGGAAATGAATTTGCTAGCTAT
GCTTTTGGGATGCCTCAGTGATATCTTTGTTTCTTGTTCTGGGGAGGATGGTTTGCTACAGGCTACAA
AGTTGGAACGGTTGTGTGAGACAAGTGAACGTGTCATTGGAGACTTGAAATTTGTTATGAGCCATG
CTATAGTTTCTAAGTATGCAACACATGAACACCGGGAGTTATCAAGATCATGGTTGACACTCTTGAC
CTTTGCTCAAGGAATGAATCCTTTAAAAAGAGAGACTGGAATCCCTATTGACGAAGAAAATGATTAC
ATGCATCTGTTCTTTGTTTTGGGTCATTCTATAGCTGTTATTCACTCTCTATTGGTTAATGGTACATATT
CTGCTGCCAGTGATGAAGAAATAGAAAATGATAGAAACGCCAAGGAAGAATTTGACAAGTGTGATG
GAGATGGAGAAAGGTATGCAAAAGTTGGAAGATTGTCTCATGAAGATTCTGTATGTACTGCGATTG
TAAGCAGCAGCTCCTTTGATAGCTCAATGGCCTCTGAAGTCCACAAAATTGATCCTTTCCATGCCCTG
CTTCCTTCTTCTGCCATATATTTGATACGCGAATGTTTGAAAGTTTTGGAGACATGCTTAGGAAATGA
TGAAGGTATATCAAAGTTTCTTTGCAAGTTGTCCTCTTCTAGCGGCAGAAACATCCCTGAAAGTAAG
ATGTCGTGGCCAAGGAGAGATTTGTTAAATGTTGAAACTGGAGGAAGTGTATCTAGTAATCTTGCCA

GTTCTAGTAGAGATCCGAGTACTGGCTTATCACCTCTATGCGGCGATATTCAAACAAATCTTAGCTTG
GATAATGTTTGTGGACCGTATGGGGTGGTTCAGACAGATGTTACAGCTGACTCTAAAAGAGTATCTT
GCAACTCTGCTGATTTGACAAAGAATGCATCAGGATTACGTATACTTGGTCTGTGTGATTGGCCGGA
CATTCACTATGATGTGAGTTCTCAGGCCATATCAGTTCATCTTCCTTTGCACCGGTTACTTTCTCTGCT
AATACAAAAAGCATTAAGGATATGTTATGGAGAATCTGCATCATACAATGGAGTCAGTATTAGTCAT
GAGATCCCACATGCAGACTTCTTCAGCTCCGTGATAGGAGATTTCCATCCTTGTGGATTTTCTGCACT
CGTTATGGAACATGTTCTACAGATTAGGGTGTTTTGTGCCCAGGTTATCGCTGGAATGTGGAAGAAG
AATGGGGATTCTGCATTAGTATCTTGTGAGTGGTATCGGTCAGTTCGTTGGTCAGAACAGGGACTTG
AGCTTGATCTATTTCTTCTCCAGTGTTGTGCTGCGTTAGCTCCCGCAGATTCTTATGTTGATAAGCTTC
TCAGTCGATTTGGGCTCTCAAGCTATCTTTCACTTAATCCGGATATAACAAATGAGTATGAACCAGTT
TTGGTCCAGGAAATGCTTGGTCTTCTGATACAGATTCTGCAAGAAAGACGATTTTGTGGTCTTTCTAC
TGCTGAAAGTTTGAGAAGAGAGATCATCTTCAAGTTAGCCACTGGAGATTTCACTCATAGTCAATTG
GTCAAATCTTTACCCCGTGACCTGTCCAAGTCGGACGAGCTCCAGGAGGTCTTAGATGACGTTTCAG
TCTATTGCAATCCATCCGGCATGAACCAGGGAAAGTATTCACTACAGTCATCTTGTTGGAAGGAATT
GGATCTCTACCACCCTCGTTGGCAGTCGAGAGACTTGCAATCTGCAGAAGAAAGATTTTCGCGTTAT
TGTGGAGTGTCTGCGTTGACCACTCAGCTTCCGAGGTGGAGAATGATCTATCCACCTCTAAAGGGGC
TTGCTAGAATAGGCACTTGCAAAGCCACATTTCAAATAATTTCATCTGCTTTATACTACGCACTACAG
AGTGGTACATCAGTTAAATCACGTGCTCCTGATGGTGTTCTCATAACTGCACTGCAGTTACTCTCGTT
ATCATTGGATATTTGCACTCAGCAGAGGCAATCTAATAGTCAGGATTGTTGCTTAGAAAATTCAATTC
CAATTCTCGAATTAGCTGGTCTGGAAATAATTGGTATAGCTCAAGGGACTGAAAAAGAAAGTTTATT
GTCTCTTCTTGTTTCGTTAATGAAGACTCGTATGGGAGATGGCCGCCATCAATTTCCAGAGCCAGGC
AGTTGCAATATTTCGTCCTGGATTGGAAATCTGCTGAAGAAGTTCAGTGCAATTGATTCTGTTTGTAT
GAACCTATTGCAAAGTCTTGCCCCCGAGGTGGTTGGTCAATCTGGCTTCGATAAAGTCATGTCAGGT
TCTACTTCTGATGAGAAGCGCAAGGCTAAAGCTAAAGAGAGGCAGGCTGCTATTATGGCAAAAATG
AAAGCTGAACAGTCAAAGTTCTTGTCTACCTTGAGTTCCAGCATGGATGATGATGATCCGAGATCTG
AATTTGAAACAAGTGATTCTGTGATGGAACATGATTCAGAAATAGCTGTCCGTGAAGTTTGCTCCCT
CTGCCATGACCCAGATTCCAAAGACCCCGTTTCTTTCTTGATTTTTCTCCAGAAATCTAAACTATTGAG
TTTTGTGGATAGAGGCCCTCCATCTTGGGATCAATGTCCACAATCCGAAAAGAAGATATCTGTGGAT
GGAGCTCCTGATCTTTTGAGAATGAATGCTTCTTCAGATAGTTTAAGAATAAGTTCTCCTCTCATGCT
GCAATTGTCAGATGATACTATCTCTGAGTCTGCTAATATGATTGAATCTATAAAAGCCCGGCTTATAG
GGAATGGTCAGACAGAGAAGAGGTCTAGCGATGGGAGGGGAAAAGATGAATCTAACATGGAATCA
TTGGAGATAGCCATGTATCAAACTGTTCGCAACAAAATTGAAAACATGATAAATCAAAGCCTTACAC
GTGTAGATCATCAACCCCATGAAGCTGAAAATTGTTCGGAGAAAAATTCTGTTGGTGGTCCATCTAC
TTTGCAAGGTAGATTTCCTGATATTCGATCAAGACAAACTTCCAGGCGTCCTGATGCTGGTTCAGAT
GGGTTTCACCCTATCGACTGTGATGGGGTTTATCTTTCATCATGTGGACATGCTGTACATCAAAGCTG
CCTTGAACGATATTTAAAATCCTTAAAGGAAAGGTCTGGCAGAAGAACTGTCTTTGAAGGTGCACAT
ATTGTGGATCTAAAAAAGAAAGAATTCCTGTGTCCTGTATGCCGCCGACTAGCCAATTCTGTATTGCC
TGAATGTCCTGGAGATTTATGTTCTGTCTCAAAGCTACAAGATAGTCCACGTACTAAGTTACGTAGA
AAAGATGCACTGCAGCCTTCTCTTTGGCTCTCTGAAGCGTTGTGTCTACTACGATCTGCTGCTGAAGT
GATCGAAGATGGTGATAGAGGTAAAACAGTTACTCCACAGGGAGATGGACCAAGAAGGAAAGATC
TTAAATCTGTCTCTAAAATGCTCTGGGATTTCTATTTTCCCAAGCCAGAGGATAAAACTCTAAAACGT
CTGTGGCTACCACCACAATCAATCGTCATGTGGGACACCCTTAAGTACTCTCTCATTTCAATGGAAAT
TGGTACTCGTTTTGCCAAGAATTCAATGCTGCCTGTCTATTGTATTGACTCCTTGTATGAAGAGTTGA
AAACCTCTAAGGGGACTATTTTGTCAGTTTTATTACGAGTGGTACAGAGCTCCAGAACAAAGAATAC
TATTCATGTTCGTCAGAGATTTGTGGGTATGAAGCATCTTGCAGAGTCTATATGCTATGGAGTTTCAA
GTAGTTCTTCGAGCAGCATATTTGGAAGTGAAGGCACAACAGGTTCATTGAAGAATATAGACCTGCT
CTGGAATCGAGCATCCGATCCAGTTCTTGCCCATGACCCATTTTCATCACTGATGTGGGCTCTCTTTT

GTCTTCCTTTTCCTTTTCTCACGTGCGAGGAATCTCTATTGTCACTTGTGCATATTTTCCACAGTGTCTC
GCTTGTTCAGACTGTAATTGCTTATTGTGCATGTCGCCCAAGTGAATTGAGTGAATTAAATTTTGGGG
AAAACCTGCTTAATGACATCTCCAACGCTTTGAGAGAATCAGGTGGTTGGGAGTATTTCAGGTCGAA
CAATATGGATTTGTCATGTGATATAAAAGACACAATCCGTAAATATAGTCTTCCTTTCTTGAGGAGAT
GTGCGTTGTTGTGGAAGCTACTGAAATCTACTCCTAGGAAGCTTCATGAAGAGTCAGATATGTTTGA
TTTGCCATCTGACCCCACCACCGACAATATGGATTTCATATACAGTCCCCAGTCTGAGCTGAATCATG
TGCAAGAATTGGAGAAAATGTTTAACATTCCACCTATAGATATCATTCTTAATGATGAACTTCTCCGT
TCTTCAACCCAAATATGGCTCCAGCATTTTCAGCGGGAATATAGAGTCAACAGAGTTAAAAGGTCCC
TTTGCATCACCCCTGTAGTTCCTTTCCAGCTAATGAAATTACCCAATCTATACCAGGACCTACTACAAA
GGTGTATAAAAAAACGTTGCGTCAACTGCACCAAAGTTATCGAAGAACCTGTATTATGTCTTCTGTG
TGGAAGTTTGTGTTCTCCCATATGGAGCCCTTGTTGCAGAGAAAGTGGTTGTCCTAATCATGCAATTA
CCTGTGGTGCTGGTACTGGTGTATTTCTTCTGATACGGCGGACCACAATTTTGCTACAAAGGTTTGCA
AGACAATCACCTTGGCCATCTCCTTACCTGGATACGTTTGGAGAAGAGGATATCGATATGATCAGAG
GAAAACGACTATACCTGAATGAAGAACGTTATGCTGCTTTAACTTACTTGGTTGGTTCTCATGGACTT
GATCGCAGTTCAAAAGTTCTCGGCCAAACCACAATTGGAGCCGTCTTACATTAAGTTAACTAtgggatc
tcccatgtctctactggtggtggtgcttctttggaattattggaaggtaaggaattgccaggtgttgctttcttatccgaaaagaaata
aattgaattgaattgaaatcgatagatcaattttttttcttttctctttccccatcctttacgctaaaataatagtttattttatttttttgaat
atttttatttatatacgtatatatagactattatttatcttttaatgattattaagatttttattaaaaaaaaattcgctcctcttttaatg
cctttatgcagttttttttttcccattcgatatttctatgttcgggttcagcgtattttaagtttaataactcgaaaattctgcgttcgttaaa
gcttgcatgcctgcaggtcgactctagaggatccccgggtaccgagctcgaattcactggccgtcgttttacaacgtcgtgactggga
aaaccctggcgttacccaacttaatcgccttgcagcacatccccctttcgccagctggcgtaatagcgaagaggcccgcaccgatcg
cccttcccaacagttgcgcagcctgaatggcgaatggcgcctgatgcggtattttctccttacgcatctgtgcggtatttcacaccgca
tatatcgctgggccattctcatgaagaatatcttgaatttattgtcatattactagttggtgtggaagtccatatatcggtgatcaatat
agtggttgacatgctggctagtcaacattgagccttttgatcatgcaaatatattacggtattttacaatcaaatatcaaacttaacta
ttgactttataacttatttaggtggtaacattcttataaaaaagaaaaaaattactgcaaaacagtactagcttttaacttgtatccta
ggttatctatgctgtctcaccatagagaatattacctatttcagaatgtatgtccatgattcgccgggtaaatacatataatacacaaa
tctggcttaataaagtctataatatatctcataaagaagtgctaaattggctagtgctatatatttttaagaaaatttcttttgactaag
tccatatcgactttgtaaaagttcactttagcatacatatattacacgagccagaaattgtaacttttgcctaaaatcacaaattgcaa
aatttaattgcttgcaaaaggtcacatgcttataatcaactttttaaaaatttaaaatactttttatttttatttttaaacataaatga
aataatttatttattgtttatgattaccgaaacataaaacctgctcaagaaaaagaaactgttttgtccttggaaaaaaagcactacc
taggagcggccaaaatgccgaggctttcatagcttaaactctttacagaaaataggcattatagatcagttcgagttttcttattcttc
cttccggttttatcgtcacagttttacagtaaataagtatcacctcttagagttcgatgataagctgtcaaacatgagaattaattcca
catgttaaaatagtgaaggagcatgttcggcacacagtggaccgaacgtggggtaagtgcactagggtccggttaaacggatctcg
cattgatgaggcaacgctaattatcaacatatagattgttatctatctgcatgaacacgaaatctttacttgacgacttgaggctgatg
gtgtttatgcaaagaaaccactgtgtttaatatgtgtcactgtttgatattactgtcagcgtagaagataatagtaaaagcggttaata
agtgtatttgagataagtgtgataaagtttttacagcgaaaagacgataaatacaagaaaatgattacgaggatacggagagagg
tatgtacatgtgtatttatatactaagctgccggcggttgtttgcaagaccgagaaaaggctagcaagaatcgggtcattgtagcgta
tgcgcctgtgaacattctcttcaacaagtttgattccattgcggtgaaatggtaaaagtcaacccccctgcgatgtatattttcctgtac
aatcaatcaaaaagccaaatgatttagcattatctttacatcttgttattttacagattttatgtttagatcttttatgcttgcttttcaaa
aggcttgcaggcaagtgcacaaacaatacttaaataaatactactcagtaataacctatttcttagcatttttgacgaaatttgctatt
ttgttagagtcttttacaccatttgtctccacacctccgcttacatcaacaccaataacgccatttaatctaagcgcatcaccaacattt
tctggcgtcagtccaccagctaacataaaatgtaagctctcggggctctcttgccttccaacccagtcagaaatcgagttccaatcca
aaagttcacctgtcccacctgcttctgaatcaaacaagggaataaacgaatgaggtttctgtgaagctgcactgagtagtatgttgc
agtcttttggaaatacgagtcttttaataactggcaaaccgaggaactcttggtattcttgccacgactcatctccatgcagttggacg
atatcaatgccgtaatcattgaccagagccaaaacatcctccttaggttgattacgaaacacgccaaccaagtatttcggagtgcct
gaactatttttatatgcttttacaagacttgaaattttccttgcaataaccgggtcaattgttctctttctattgggcacacatataatac
ccagcaagtcagcatcggaatctagtgcacattctgcggcctctgtgctctgcaagccgcaaactttcaccaatggaccagaactac

ctgtgaaattaataacagacatactccaagctgcctttgtgtgcttaatcacgtatactcacgtgctcaatagtcaccaatgccctccc
tcttggccctctccttttctttttcgaccgaattaattcttgaagacgaaagggcctcgtgatacgcctatttttataggttaatgtcat
gataataatggtttcttagacgtcaggtggcacttttcggggaaatgtgcgcggaacccctatttgtttatttttctaaatacattcaaa
tatgtatccgctcatgagacaataaccctgataaatgcttcaataatattgaaaaaggaagagtatgagtattcaacatttccgtgtc
gcccttattcccttttttgcggcattttgccttcctgtttttgctcacccagaaacgctggtgaaagtaaaagatgctgaagatcagttg
ggtgcacgagtgggttacatcgaactggatctcaacagcggtaagatccttgagagttttcgccccgaagaacgttttccaatgatg
agcacttttaaagttctgctatgtggcgcggtattatcccgtattgacgccgggcaagagcaactcggtcgccgcatacactattctc
agaatgacttggttgagtactcaccagtcacagaaaagcatcttacggatggcatgacagtaagagaattatgcagtgctgccata
accatgagtgataacactgcggccaacttacttctgacaacgatcggaggaccgaaggagctaaccgcttttttgcacaacatggg
ggatcatgtaactcgccttgatcgttgggaaccggagctgaatgaagccataccaaacgacgagcgtgacaccacgatgcctgtag
caatggcaacaacgttgcgcaaactattaactggcgaactacttactctagcttcccggcaacaattaatagactggatggaggcgg
ataaagttgcaggaccacttctgcgctcggcccttccggctggctggtttattgctgataaatctggagccggtgagcgtgggtctcg
cggtatcattgcagcactggggccagatggtaagccctcccgtatcgtagttatctacacgacggggagtcaggcaactatggatga
acgaaatagacagatcgctgagataggtgcctcactgattaagcattggtaactgtcagaccaagtttactcatatatactttagatt
gatttaaaacttcatttttaatttaaaaggatctaggtgaagatcctttttgataatctcatgaccaaaatcccttaacgtgagttttcg
ttccactgagcgtcagaccccgtagaaaagatcaaaggatcttcttgagatcctttttttctgcgcgtaatctgctgcttgcaaacaaa
aaaaccaccgctaccagcggtggtttgtttgccggatcaagagctaccaactctttttccgaaggtaactggcttcagcagagcgca
gataccaaatactgtccttctagtgtagccgtagttaggccaccacttcaagaactctgtagcaccgcctacatacctcgctctgcta
atcctgttaccagtggctgctgccagtggcgataagtcgtgtcttaccgggttggactcaagacgatagttaccggataaggcgcag
cggtcgggctgaacggggggttcgtgcacacagcccagcttggagcgaacgacctacaccgaactgagatacctacagcgtgagc
tatgagaaagcgccacgcttcccgaagggagaaaggcggacaggtatccggtaagcggcagggtcggaacaggagagcgcacg
agggagcttccagggggaaacgcctggtatctttatagtcctgtcgggtttcgccacctctgacttgagcgtcgatttttgtgatgctc
gtcaggggggcggagcctatggaaaaacgccagcaacgcggcctttttacggttcctggccttttgctggccttttgctcacatgttct
ttcctgcgttatcccctgattctgtggataaccgtattaccgcctttgagtgagctgataccgctcgccgcagccgaacgaccgagcg
cagcgagtcagtgagcgaggaagcggaaga
