# Supplementary material for: A Yeast-Based Functional Assay to Study Plant N-Degron – N-Recognin Interactions
Source: Front Plant Sci. 2022 Jan 7;12:806129. doi: 10.3389/fpls.2021.806129 (PMC8777003; doi:10.3389/fpls.2021.806129)
Supplement: Supplementary file 7 [file Data_Sheet_5.pdf]

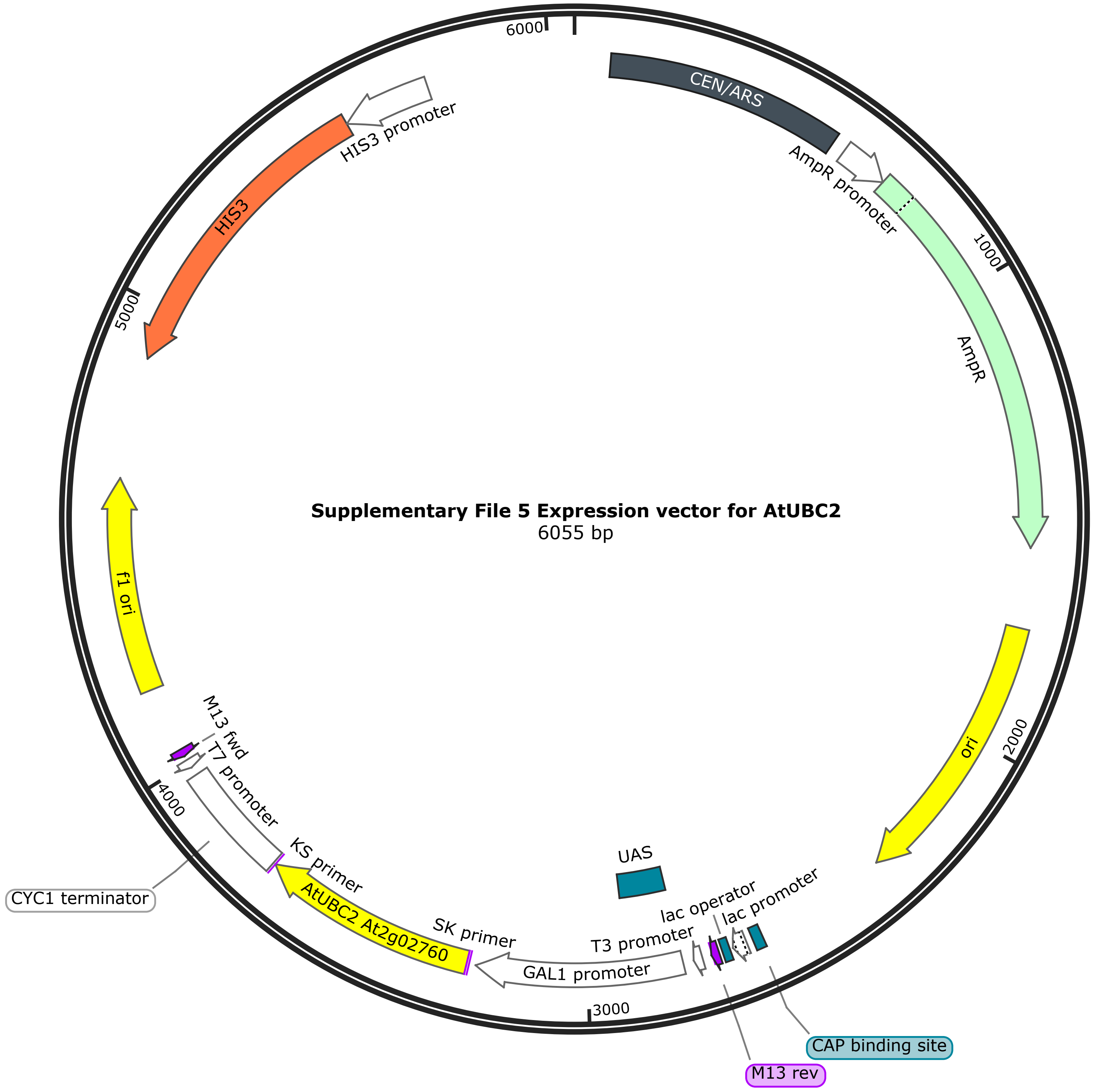

Supplementary File 5 Expression vector for AtUBC2
6055 bp
6000
CEN/ARS
AmpR promoter
1000
AmpR
ori
2000
UAS
lac operator
lac promoter
CAP binding site
M13 rev
T3 promoter
GAL1 promoter
3000
SK primer
AtUBC2 At2g02760
KS primer
T7 promoter
M13 fwd
CYC1 terminator
4000
f1 ori
5000
HIS3
HIS3 promoter

gacgaaagggcctcgtgatacgcctatttttataggttaatgtcatgataataatggtttcttagggtccttttcatcacgtgctataaa
aataattataatttaaatttttaatataaatatataaattaaaaatagaaagtaaaaaaagaaattaaagaaaaaatagtttttgtt
ttccgaagatgtaaaagactctagggggatcgccaacaaatactacctttatcttgctcttcctgctctcaggtattaatgccgaatt
gtttcatcttgtctgtgtagaagaccacacacgaaaatcctgtgattttacattttacttatcgttaatcgaatgtatatctatttaatct
gcttttcttgtctaataaatatatatgtaaagtacgctttttgttgaaattttttaaacctttgtttatttttttttcttcattccgtaactctt
ctaccttctttatttactttctaaaatccaaatacaaaacataaaaataaataaacacagagtaaattcccaaattattccatcattaa
aagatacgaggcgcgtgtaagttacaggcaagcgatccgtcaggtggcacttttcggggaaatgtgcgcggaacccctatttgttta
tttttctaaatacattcaaatatgtatccgctcatgagacaataaccctgataaatgcttcaataatattgaaaaaggaagagtatga
gtattcaacatttccgtgtcgcccttattcccttttttgcggcattttgccttcctgtttttgctcacccagaaacgctggtgaaagtaaa
agatgctgaagatcagttgggtgcacgagtgggttacatcgaactggatctcaacagcggtaagatccttgagagttttcgccccga
agaacgttttccaatgatgagcacttttaaagttctgctatgtggcgcggtattatcccgtattgacgccgggcaagagcaactcggt
cgccgcatacactattctcagaatgacttggttgagtactcaccagtcacagaaaagcatcttacggatggcatgacagtaagaga
attatgcagtgctgccataaccatgagtgataacactgcggccaacttacttctgacaacgatcggaggaccgaaggagctaaccg
cttttttgcacaacatgggggatcatgtaactcgccttgatcgttgggaaccggagctgaatgaagccataccaaacgacgagcgtg
acaccacgatgcctgtagcaatggcaacaacgttgcgcaaactattaactggcgaactacttactctagcttcccggcaacaattaa
tagactggatggaggcggataaagttgcaggaccacttctgcgctcggcccttccggctggctggtttattgctgataaatctggagc
cggtgagcgtgggtctcgcggtatcattgcagcactggggccagatggtaagccctcccgtatcgtagttatctacacgacggggag
tcaggcaactatggatgaacgaaatagacagatcgctgagataggtgcctcactgattaagcattggtaactgtcagaccaagttta
ctcatatatactttagattgatttaaaacttcatttttaatttaaaaggatctaggtgaagatcctttttgataatctcatgaccaaaatc
ccttaacgtgagttttcgttccactgagcgtcagaccccgtagaaaagatcaaaggatcttcttgagatcctttttttctgcgcgtaatc
tgctgcttgcaaacaaaaaaaccaccgctaccagcggtggtttgtttgccggatcaagagctaccaactctttttccgaaggtaactg
gcttcagcagagcgcagataccaaatactgttcttctagtgtagccgtagttaggccaccacttcaagaactctgtagcaccgcctac
atacctcgctctgctaatcctgttaccagtggctgctgccagtggcgataagtcgtgtcttaccgggttggactcaagacgatagttac
cggataaggcgcagcggtcgggctgaacggggggttcgtgcacacagcccagcttggagcgaacgacctacaccgaactgagata
cctacagcgtgagctatgagaaagcgccacgcttcccgaagggagaaaggcggacaggtatccggtaagcggcagggtcggaac
aggagagcgcacgagggagcttccagggggaaacgcctggtatctttatagtcctgtcgggtttcgccacctctgacttgagcgtcg
atttttgtgatgctcgtcaggggggcggagcctatggaaaaacgccagcaacgcggcctttttacggttcctggccttttgctggcctt
ttgctcacatgttctttcctgcgttatcccctgattctgtggataaccgtattaccgcctttgagtgagctgataccgctcgccgcagcc
gaacgaccgagcgcagcgagtcagtgagcgaggaagcggaagagcgcccaatacgcaaaccgcctctccccgcgcgttggccga
ttcattaatgcagctggcacgacaggtttcccgactggaaagcgggcagtgagcgcaacgcaattaatgtgagttagctcactcatt
aggcaccccaggctttacactttatgcttccggctcgtatgttgtgtggaattgtgagcggataacaatttcacacaggaaacagcta
tgaccatgattacgccaagcgcgcaattaaccctcactaaagggaacaaaagctggagctctagtacggattagaagccgccgag
cgggtgacagccctccgaaggaagactctcctccgtgcgtcctcgtcttcaccggtcgcgttcctgaaacgcagatgtgcctcgcgcc
gcactgctccgaacaataaagattctacaatactagcttttatggttatgaagaggaaaaattggcagtaacctggccccacaaacc
ttcaaatgaacgaatcaaattaacaaccataggatgataatgcgattagttttttagccttatttctggggtaattaatcagcgaagc
gatgatttttgatctattaacagatatataaatgcaaaaactgcataaccactttaactaatactttcaacattttcggtttgtattactt
cttattcaaatgtaataaaagtatcaacaaaaaattgttaatatacctctatactttaacgtcaaggagaaaaAACCCCGGATT
CTAGATGTCGACTCCAGCGAggaagagattgatgagggatttcaagaggttgcagcaagacccacctgcaggaattagtg
gtgctccacaagacaacaatatcatgttgtggaatgctgttatattcgggcctgatgataccccttgggatggaggtactttcaaattg
tcactgcagttttcggaagattatccaaataaaccaccaacagtgcggtttgtttcacggatgttccatccaaacatttatgctgatgg
gagtatatgcttggacattctccaaaaccaatggagtccaatatacgatgtcgctgctatactaacctccattcagtcattgctatgtg
atcctaatccgaattctcctgcaaattcggaagctgcacgaatgttcagtgaaagcaagcgcgagtacaacagaagagtccgcgag
gttgtcgaacaaaGCTGGACTGCCGACTAGTCGAGTCATGTAATTagttatgtcacgcttacattcacgccctccccc
cacatccgctctaaccgaaaaggaaggagttagacaacctgaagtctaggtccctatttatttttttatagttatgttagtattaagaa
cgttatttatatttcaaatttttcttttttttctgtacagacgcgtgtacgcatgtaacattatactgaaaaccttgcttgagaaggttttg
ggacgctcgaaggctttaatttgcggccggtacccaattcgccctatagtgagtcgtattacgcgcgctcactggccgtcgttttacaa
cgtcgtgactgggaaaaccctggcgttacccaacttaatcgccttgcagcacatccccctttcgccagctggcgtaatagcgaagag

gcccgcaccgatcgcccttcccaacagttgcgcagcctgaatggcgaatggacgcgccctgtagcggcgcattaagcgcggcgggt
gtggtggttacgcgcagcgtgaccgctacacttgccagcgccctagcgcccgctcctttcgctttcttcccttcctttctcgccacgttc
gccggctttccccgtcaagctctaaatcgggggctccctttagggttccgatttagtgctttacggcacctcgaccccaaaaaacttga
ttagggtgatggttcacgtagtgggccatcgccctgatagacggtttttcgccctttgacgttggagtccacgttctttaatagtggact
cttgttccaaactggaacaacactcaaccctatctcggtctattcttttgatttataagggattttgccgatttcggcctattggttaaaa
aatgagctgatttaacaaaaatttaacgcgaattttaacaaaatattaacgcttacaatttcctgatgcggtattttctccttacgcatc
tgtgcggtatttcacaccgcatagatccgtcgagttcaagagaaaaaaaaagaaaaagcaaaaagaaaaaaggaaagcgcgcct
cgttcagaatgacacgtatagaatgatgcattaccttgtcatcttcagtatcatactgttcgtatacatacttactgacattcataggta
tacatatatacacatgtatatatatcgtatgctgcagctttaaataatcggtgtcactacataagaacacctttggtggagggaacat
cgttggtaccattgggcgaggtggcttctcttatggcaaccgcaagagccttgaacgcactctcactacggtgatgatcattcttgcct
cgcagacaatcaacgtggagggtaattctgctagcctctgcaaagctttcaagaaaatgcgggatcatctcgcaagagagatctcct
actttctccctttgcaaaccaagttcgacaactgcgtacggcctgttcgaaagatctaccaccgctctggaaagtgcctcatccaaag
gcgcaaatcctgatccaaacctttttactccacgcacggcccctagggcctctttaaaagcttgaccgagagcaatcccgcagtcttc
agtggtgtgatggtcgtctatgtgtaagtcaccaatgcactcaacgattagcgaccagccggaatgcttggccagagcatgtatcat
atggtccagaaaccctatacctgtgtggacgttaatcacttgcgattgtgtggcctgttctgctactgcttctgcctctttttctgggaag
atcgagtgctctatcgctaggggaccaccctttaaagagatcgcaatctgaatcttggtttcatttgtaatacgctttactagggctttc
tgctctgtcatctttgccttcgtttatcttgcctgctcatttttagtatattcttcgaagaaatcacattactttatataatgtataattcat
tatgtgataatgccaatcgctaagaaaaaaaaagagtcatccgctaggtggaaaaaaaaaaatgaaaatcattaccgaggcataa
aaaaatatagagtgtactagaggaggccaagagtaatagaaaaagaaaattgcgggaaaggactgtgttatgacttccctgacta
atgccgtgttcaaacgatacctggcagtgactcctagcgctcaccaagctcttaaaacgggaatttatggtgcactctcagtacaatc
tgctctgatgccgcatagttaagccagccccgacacccgccaacacccgctgacgcgccctgacgggcttgtctgctcccggcatcc
gcttacagacaagctgtgaccgtctccgggagctgcatgtgtcagaggttttcaccgtcatcaccgaaacgcgcga
