# Supplementary material for: A Yeast-Based Functional Assay to Study Plant N-Degron – N-Recognin Interactions
Source: Front Plant Sci. 2022 Jan 7;12:806129. doi: 10.3389/fpls.2021.806129 (PMC8777003; doi:10.3389/fpls.2021.806129)
Supplement: Supplementary file 8 [file Data_Sheet_6.pdf]

Supplementary File 6.
Numerical values depicted in Figures 1, 2, 4, 5, 7 and Supplementary Figure 2.

Figure 1A	Arg-βGal relative value 0.6 s.dev 0.4 n=15<br>Val-βGal relative value 100 s.dev 34.1 n=13
Figure 1B:	Arg-βGal relative value 58 s.dev 21.5 n=15<br>Val-βGal relative value 100 s.dev 30.3 n=13
Figure 1C:	Arg-βGal relative value 79.9 s.dev 10 n=9<br>Val-βGal relative value 100 s.dev 12.25 n=10
Figure 1D:	Arg-βGal relative value 12.3 s.dev 3.7 n=10<br>Val-βGal relative value 100 s.dev 17.2 n=10
Figure 2A	Arg-GFP 2 hr relative value 58.9 s.dev 6.9 n=15<br>Val-GFP 2 hr relative value 100 s.dev 10.5 n=15<br>100% corresponded to a count of 268500 per cell density unit (OD600) on the plate reader.<br>Arg-GFP 24 hr relative value 49.6 s.dev 2.9 n=15<br>Val-GFP 24 hr relative value 100 s.dev 4 n=15<br>100% corresponded to a count of 267800 per cell density unit (OD600) on the plate reader.
Figure 2B	Arg-GFP 2 hr relative value 93.5 s.dev 13.8 n=14<br>Val-GFP 2 hr relative value 100 s.dev 15.8 n=15<br>100% corresponded to a count of 339600 per cell density unit (OD600) on the plate reader.<br>Arg-GFP 24 hr relative value 89.3 s.dev 22.1 n=15<br>Val-GFP 24 hr relative value 100 s.dev 5.7 n=15<br>100% corresponded to a count of 409900 per cell density unit (OD600) on the plate reader.
Figure 2C	Arg-GFP 2 hr relative value 100.3 s.dev 29.8 n=15<br>Val-GFP 2 hr relative value 100 s.dev 8.6 n=15<br>100% corresponded to a count of 522600 per cell density unit (OD600) on the plate reader.<br>Arg-GFP 24 hr relative value 95.9 s.dev 8.1 n=15<br>Val-GFP 24 hr relative value 100 s.dev 6.6 n=15<br>100% corresponded to a count of 354300 per cell density unit (OD600) on the plate reader.
Figure 2D	Arg-GFP 2 hr relative value 91.2 s.dev 10.2 n=15<br>Val-GFP 2 hr relative value 100 s.dev 7.4 n=15<br>100% corresponded to a count of 456300 per cell density unit (OD600)

on the plate reader.
Arg-GFP 24 hr relative value 44.5 s.dev 7.0 n=15
Val-GFP 24 hr relative value 100 s.dev 7.8 n=15
100% corresponded to a count of 297500 per cell density unit (OD600) on the plate reader.

Figure 4A Arg-HRE2-GFP PRT6+AtUBC2 relative value 44.4 s.dev 29.8 n=12
Ala-HRE2-GFP PRT6+AtUBC2 relative value 100 s.dev 31.7 n=12
100% corresponded to a count of 58730 per cell density unit (OD600) on the plate reader.
Arg-HRE2-GFP PRT6 only relative value 112 s.dev 23.9 n=12
Ala-HRE2-GFP PRT6 only relative value 100 s.dev 15.9 n=12
100% corresponded to a count of 94620 per cell density unit (OD600) on the plate reader.

Figure 4B Arg-ZPR2-GFP PRT6+AtUBC2 relative value 65.8 s.dev 23.5 n=12
Ala-ZPR2-GFP PRT6+AtUBC2 relative value 100 s.dev 28.5 n=12
100% corresponded to a count of 41660 per cell density unit (OD600) on the plate reader.
Arg-ZPR2-GFP PRT6 only relative value 76.4 s.dev 18.1 n=12
Ala-ZPR2-GFP PRT6 only relative value 100 s.dev 37 n=12
100% corresponded to a count of 115060 per cell density unit (OD600) on the plate reader.

Figure 5A Arg-BBX31-GFP PRT6+AtUBC2 relative value 70.4 s.dev 14.7 n=21
Ala-BBX31-GFP PRT6+AtUBC2 relative value 100 s.dev 13.1 n=21
100% corresponded to a count of 157500 per cell density unit (OD600) on the plate reader.
Arg-BBX31-GFP PRT6 only relative value 106.8 s.dev 15.1 n=12
Ala-BBX31-GFP PRT6 only relative value 100 s.dev 12.4 n=12
100% corresponded to a count of 172100 per cell density unit (OD600) on the plate reader.

Figure 5B Arg-BBX30-GFP PRT6+AtUBC2 relative value 72.3 s.dev 37.3 n=12
Ala-BBX30-GFP PRT6+AtUBC2 relatve value 100 s.dev 13.2 n=9
100% corresponded to a count of 44270 per cell density unit (OD600) on the plate reader.
Arg-BBX30-GFP PRT6 only relative value 92.6 s.dev 22.2 n=12
Ala-BBX30-GFP PRT6 only relative value 100 s.dev 27.7 n=12
100% corresponded to a count of 88640 per cell density unit (OD600) on the plate reader.

Figure 5C Arg-bHLH38-GFP PRT6+AtUBC2 relative value 78.5 s.dev 19.5 n=27
Ala-bHLH38-GFP PRT6+AtUBC2 relative value 100 s.dev 23.9 n=27
100% corresponded to a count of 43550 per cell density unit (OD600)

on the plate reader.
Arg-bHLH38-GFP PRT6 only relative value 81.7 s.dev 26.3 n=18
Ala-bHLH38-GFP PRT6 only relative value 100 s.dev 15.6 n=18
100% corresponded to a count of 81800 per cell density unit (OD600) on the plate reader.

Figure 7A

Arg-RIN4III-GFP PRT6+AtUBC2 relative value 73.6 s.dev 17.6 n=27
Ala-RIN4III-GFP PRT6+AtUBC2 relative value 100 s.dev 26.6 n=27
100% corresponded to a count of 290500 per cell density unit (OD600) on the plate reader.
Arg-RIN4III-GFP PRT6 only relative value 81.7 s.dev 11.8 n=18
Ala-RIN4III-GFP PRT6 only relative value 100 s.dev 16.8 n=18
100% corresponded to a count of 337400 per cell density unit (OD600) on the plate reader.
Arg-RIN4III-GFP no PRT6 or AtUBC2 relative value 94.5 s.dev 28.7 n=14
Ala-RIN4III-GFP no PRT6 or AtUBC2 relative value 100 s.dev 23.4 n=15
100% corresponded to a count of 610100 per cell density unit (OD600) on the plate reader.

Supplementary Figure 2A

Arg-VRN2-GFP PRT6+AtUBC2 relative value 85.7 s.dev 40.1 n=12
Ala-VRN2-GFP PRT6+AtUBC2 relative value 100 s.dev 22.6 n=12
100% corresponded to a count of 10490 per cell density unit (OD600) on the plate reader.
Arg-VRN2-GFP PRT6 only relative value 110.8 s.dev 23 n=11
Ala-VRN2-GFP PRT6 only relative value 100 s.dev 16.5 n=12
100% corresponded to a count of 15070 per cell density unit (OD600) on the plate reader.
